# Functional Specialization in *Vibrio cholerae* Diguanylate Cyclases: Distinct Modes of Motility Suppression and c-di-GMP Production

**DOI:** 10.1128/mBio.00670-19

**Published:** 2019-04-23

**Authors:** David Zamorano-Sánchez, Wujing Xian, Calvin K. Lee, Mauro Salinas, Wiriya Thongsomboon, Lynette Cegelski, Gerard C. L. Wong, Fitnat H. Yildiz

**Affiliations:** a Department of Microbiology and Environmental Toxicology, University of California, Santa Cruz, Santa Cruz, California, USA; b Department of Bioengineering, University of California, Los Angeles, Los Angeles, California, USA; c Department of Chemistry and Biochemistry, University of California, Los Angeles, Los Angeles, California, USA; d California Nano Systems Institute, University of California, Los Angeles, Los Angeles, California, USA; e Department of Chemistry, Stanford University, Stanford, California, USA; University of Washington; Loyola University Chicago; University of Queensland

**Keywords:** biofilm, motility, *Vibrio cholerae*, c-di-GMP

## Abstract

Vibrio cholerae biofilm formation and associated motility suppression are correlated with increased concentrations of cyclic diguanylate monophosphate (c-di-GMP), which are in turn driven by increased levels and/or activity of diguanylate cyclases (DGCs). To further our understanding of how c-di-GMP modulators in V. cholerae individually and collectively influence motility with cellular resolution, we determined how DGCs CdgD and CdgH impact intracellular c-di-GMP levels, motility, and biofilm formation. Our results indicated that CdgH strongly influences swim speed distributions; cells in which *cdgH* was deleted had higher average swim speeds than wild-type cells. Furthermore, our results suggest that CdgD, rather than CdgH, is the dominant DGC responsible for postattachment c-di-GMP production in biofilms. Lipopolysaccharide (LPS) biosynthesis genes were found to be extragenic bypass suppressors of the motility phenotypes of strains Δ*cdgD* and Δ*cdgH*. We compared the motility regulation mechanism of the DGCs with that of Gmd, an LPS O-antigen biosynthesis protein, and discovered that comodulation of c-di-GMP levels by these motility effectors can be positively or negatively cooperative rather than simply additive. Taken together, these results suggest that different environmental and metabolic inputs orchestrate DGC responses of V. cholerae via c-di-GMP production and motility modulation.

## Abstract (precis)

Cyclic diguanylate monophosphate (c-di-GMP) is a broadly conserved bacterial signaling molecule that affects motility, biofilm formation, and virulence. Although it has been known that high intracellular concentrations of c-di-GMP correlate with motility suppression and biofilm formation, how the 53 predicted c-di-GMP modulators in Vibrio cholerae collectively influence motility is not understood in detail. Here we used a combination of plate assays and single-cell tracking methods to correlate motility and biofilm formation outcomes with specific enzymes involved in c-di-GMP synthesis in Vibrio cholerae, the causative agent of the disease cholera.

## INTRODUCTION

The ability of microorganisms to form biofilms, which are surface-attached communities of microorganisms encased in a matrix of exopolysaccharides, proteins, and nucleic acids, enhances microbial fitness (1). Biofilm formation begins with attachment of motile planktonic cells to surfaces and subsequent commitment to stable surface association. Microbial growth and matrix production on surfaces result in formation of microcolonies and eventually in formation of mature biofilms (1, 2). Different aspects of biofilm formation (e.g., cell surface interactions, biofilm matrix production, biofilm maturation, and dispersal) are controlled by various regulatory circuitries (2). The nucleotide-based intracellular signaling molecule cyclic diguanylate monophosphate (c-di-GMP) is a key component of biofilm regulatory circuitries in a diverse group of microorganisms. Cellular c-di-GMP levels are controlled by the competing activities of diguanylate cyclases (DGCs), which produce c-di-GMP, and phosphodiesterases, which degrade c-di-GMP (3, 4). The abundances of DGCs and phosphodiesterases differ among bacteria (3). The spectrum of cellular processes targeted by DGCs and phosphodiesterases and the specific roles that they play during different stages of biofilm formation are not fully understood. The paradigm of c-di-GMP signaling is also evolving; recent evidence obtained from different model organisms has made it clear that c-di-GMP metabolizing enzymes can act independently in insulated signaling circuitries or cascades (5, 6).

Biofilm formation is a critical component of the infection cycle of the human pathogen Vibrio cholerae, increasing infectivity and environmental survival of the pathogen (2, 7, 8). V. cholerae biofilm formation begins when motile cells encounter a surface and attach via the type IV mannose-sensitive hemagglutinin (MSHA) pilus (9, 10). During early stages of biofilm formation, inhibition of flagellar function and repression of flagellar production are thought to be necessary to stabilize V. cholerae cell-surface attachment. Production of biofilm matrix components *Vibrio* polysaccharide (VPS) and matrix proteins, predominantly RbmA, RbmC, and Bap,-I is required for microcolony and mature biofilm formation (11–13). MSHA pilus production, flagellum production, and biofilm matrix production are all controlled by regulatory circuitries involving c-di-GMP (14–21). Thus, in V. cholerae, c-di-GMP is a central regulatory factor governing surface attachment and biofilm formation (4).

The V. cholerae genome contains 53 proteins with domains known to be involved in c-di-GMP metabolism (https://www.ncbi.nlm.nih.gov/Complete_Genomes/c-di-GMP.html). The analysis of the amino acid sequences of these proteins revealed that 28 proteins have conserved GGDEF domains, 16 proteins have conserved EAL domains, 4 proteins contain tandem conserved GGDEF and EAL domains, and 5 proteins have conserved HD-GYP domains (although activity has been demonstrated for only 4 [22]). Only a subset of these proteins impact motility (as measured by soft agar motility assays), biofilm formation, or both (23–26). Our earlier work identified DGCs CdgD and CdgH as regulators of motility via soft agar motility assays (23–26). CdgD harbors a GGDEF domain with the conserved residues required for catalytic function, although its enzymatic activity remains to be tested; mutants lacking CdgD have markedly increased swimming motility and delayed initial surface attachment (23–26). CdgH has a conserved cytoplasmic GGDEF domain, and it functions as a DGC (25, 27, 28); mutants lacking *cdgH* have increased motility as well as decreased VPS production and biofilm formation (25, 26, 28). Although it is clear that these DGCs influence motility in some manner, the molecular mechanisms of c-di-GMP-mediated motility repression remain unclear. In this study, we analyzed the contribution of CdgD and CdgH in controlling the transition from motility to biofilm formation. In searching for suppressors of the motility phenotype of CdgD, we found that mutants deficient in O-antigen biosynthesis were affected in motility in soft agar plates. To further investigate how CdgD, CdgH, and lipopolysaccharide (LPS) production (using GDP-mannose 4,6-dehydratase [Gmd] as a representative O-antigen biosynthesis protein) impacts motility, we characterized the motility-to-biofilm transition using high-speed single-cell tracking. Our results showed that the DGCs impacted motility by changing swim speed distributions, CdgH having the strongest effect. Importantly, the single-cell tracking studies showed that the DGCs and Gmd impacted cellular behavior via completely different channels of activity, the latter influencing motility by changing the residence time spent on or near a surface.

We also found a striking difference in the contributions of the DGCs and Gmd to the overall levels of c-di-GMP production during the initial stages of biofilm formation. Using a fluorescent c-di-GMP reporter, we determined the evolution of c-di-GMP accumulation on surface-attached V. cholerae cells. We found that CdgD and CdgH contribute to postlanding c-di-GMP production with different intensities and time evolutions. A mutant lacking *gmd* accumulated more intracellular c-di-GMP than wild-type (WT) cells, had a higher rate of biomass increase, and formed thicker biofilms.

In summary, by using single-cell tracking at high speed and measuring c-di-GMP production dynamics via a biosensor in time-lapse microscopy, we showed that comodulation of c-di-GMP levels by CdgD, CdgH, and Gmd can be positively or negatively cooperative rather than simply additive. Taken together, these results suggest that there is a “division of labor” between V. cholerae DGCs that orchestrates responses of cells to environmental and metabolic inputs.

## RESULTS

### Transposon insertions in O-antigen biosynthesis genes suppress the hypermotile phenotype of Δ*cdgD*.

V. cholerae lacking the DGC CdgD exhibited a marked increase in motility in a lysogeny broth (LB) soft agar motility assay in comparison to WT bacteria (23, 26). To determine if the motility phenotype of strain Δ*cdgD* is due to its inability to produce c-di-GMP, we performed complementation assays with a strain carrying a gene coding for WT CdgD and with a strain that expresses a variant that encodes a degenerate active site (AAEEF). Ectopic expression of *cdgD* but not a *cdgD*^AAEEF^ site repressed motility to ∼50% of WT levels (see Fig. S1 in the supplemental material). This finding suggests that CdgD is capable of c-di-GMP synthesis and that this activity is important for its role as a motility repressor.

10.1128/mBio.00670-19.2 FIG S1 A conserved GGDEF domain is required for Δ*cdgD* complementation. The bar graph presents means and standard deviations of the diameters of migration of the WT strain and the Δ*cdgD*, Δ*cdgD*-pBAD (empty plasmid), Δ*cdgD*-p*cdgD*, Δ*cdgD*-p*cdgD*^AAEEF^, Δ*cdgH*-pBAD, and Δ*cdgH*-p*cdgH* mutants in plate motility assays. Means were compared to WT data using analysis of variance (ANOVA) and Dunnett's multiple-comparison test. Mean differences with an adjusted *P* value of ≤0.05 were deemed significant. ****, *P* ≤ 0.0001. Download FIG S1, TIF file, 0.4 MB. Copyright © 2019 Zamorano-Sánchez et al. 2019 Zamorano-Sánchez et al. This content is distributed under the terms of the Creative Commons Attribution 4.0 International license.

To begin investigating the mechanism by which CdgD impacts motility, we performed transposon mutagenesis in the Δ*cdgD* strain and screened 8,217 mutants for those with WT motility in an LB soft agar motility assay. In this assay, strains that are normal with respect to both motility and chemotaxis form an expanded zone of motility, whereas strains with changes in either property have altered expansion sizes.

Transposon insertion sites from 34 mutants with altered motility without impact on growth were identified (see Table S1 in the supplemental material). Twelve of these insertions were found in genes involved in LPS biosynthesis. The majority of the mutants had insertions into the genes required for perosamine biosynthesis and O-antigen biosynthesis, although one insertion was found in gene VC0239; these genes form part of a genetic locus that contains genes involved in LPS biosynthesis (Fig. S2A and B). We also identified an insertion in *galU*, a gene that encodes a UTP-glucose-1-phosphate uridylyltransferase known to be involved in LPS and VPS biosynthesis (29). These observations suggest that O-antigen biosynthesis plays a role in modulating motility in V. cholerae.

10.1128/mBio.00670-19.3 FIG S2 Multiple extragenic suppressors of hypermotility in the Δ*cdgD* mutant were present in LPS biosynthesis loci. (A) Schematic representation of LPS biosynthesis loci with transposon insertions in our forward genetics approach. Gene loci that code for proteins that participate in LPS biosynthesis are color coded in accordance with their predicted activity. Black inverted triangles represent transposon insertion events in the corresponding open reading frame. (B) The reported structure and sugar composition of LPS from V. cholerae O1 are shown. The LPS components that were affected by the in-frame deletions used in this study are marked with a rectangle. (C) LPS profile of the indicated genetic backgrounds that represented derivatives of V. cholerae O1 El Tor A1552. The LPS standard is from Escherichia coli serotype 055:B5 (Component B) (20 µg). Download FIG S2, TIF file, 1.1 MB. Copyright © 2019 Zamorano-Sánchez et al. 2019 Zamorano-Sánchez et al. This content is distributed under the terms of the Creative Commons Attribution 4.0 International license.

10.1128/mBio.00670-19.7 TABLE S1 List of transposon insertion mutants. Download Table S1, PDF file, 0.02 MB. Copyright © 2019 Zamorano-Sánchez et al. 2019 Zamorano-Sánchez et al. This content is distributed under the terms of the Creative Commons Attribution 4.0 International license.

### An O-antigen-deficient strain shows impaired motility in soft agar motility assays.

The O-antigen of V. cholerae is composed of 12 to 18 tetronate acylated perosamine repeating units (30–33). Previous studies revealed that mutations in genes predicted to participate in perosamine biosynthesis (VC0241 to VC0244) result in loss of O-antigen and associated phenotypes such as sensitivity to bacteriophages K139 and VP4 (34–36). To analyze the role of O-antigen biosynthesis in motility regulation, biofilm formation, and c-di-GMP signaling, we generated the following strains likely to be defective in O-antigen production: a strain lacking *gmd* (VC0243), which encodes a glucose mannose dehydratase that is predicted to catalyze the conversion of GDP-mannose to GDP-4-keto-6-deoxy-d-mannose (37), and a strain lacking *wavA* (VC0223), a putative heptosyl III transferase involved in LPS biosynthesis that has been previously shown to be necessary for O-antigen attachment (38). We confirmed that deletion of *gmd* and *wavA* resulted in the absence of O-antigen (Fig. S2C). Deletion of *gmd* did not affect the growth rate; however, deletion of *wavA* resulted in a lower growth rate than that of the WT strain (Fig. S3). We also observed that the Δ*gmd* strain autoaggregated (the autoaggregated cells could be dispersed by agitation), whereas the Δ*wavA* strain remained in suspension (Fig. S4). These observations indicate that the absence of either *gmd* or *wavA*, each of which affects different components of the LPS, has contrasting consequences for the cell surface properties of V. cholerae.

10.1128/mBio.00670-19.4 FIG S3 Deletion of *wavA*, but not deletion of *gmd*, affects the growth rate of V. cholerae. The growth curves of the WT, Δ*gmd*, and Δ*wavA* strains were obtained by measuring the optical density (OD) at 600 nm of shaking cultures (200 rpm) after 0,1, 2, 3, 4, 5, 6, 7, 8, and 23 h of growth in LB broth at 30°C. The points and error bars in the graph represent the averages and standard deviations of results from at least three independent biological replicates for each genetic background. Download FIG S3, TIF file, 0.3 MB. Copyright © 2019 Zamorano-Sánchez et al. 2019 Zamorano-Sánchez et al. This content is distributed under the terms of the Creative Commons Attribution 4.0 International license.

10.1128/mBio.00670-19.5 FIG S4 Null mutants in *gmd* autoaggregate in liquid culture. The image is representative of liquid cultures of the WT strain and null mutants in *cdgD*, *wavA*, and *gmd*. Grown cultures were incubated at room temperature under static conditions. Pictures were taken after 0 and 2 h. Download FIG S4, TIF file, 1.0 MB. Copyright © 2019 Zamorano-Sánchez et al. 2019 Zamorano-Sánchez et al. This content is distributed under the terms of the Creative Commons Attribution 4.0 International license.

**FIG 1:**
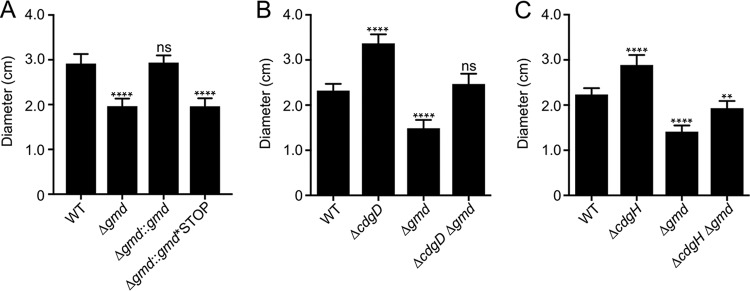
Deletion of *gmd*, *cdgD*, or *cdgH* affects motility in soft agar plates. (A to C) Bar graphs of the means and standard deviations of the diameters of migration of the (A) WT, Δ*gmd*, Δ*gmd*::*gmd*, and Δ*gmd*::gmd*STOP strains; (B) WT, Δ*cdgD*, Δ*gmd*, and Δ*cdgD* Δ*gmd* strains; and (C) WT, Δ*cdgH*, Δ*gmd*, and Δ*cdgH* Δ*gmd* strains in plate motility assays. Means were compared to the WT data using analysis of variance (ANOVA) and Dunnett's multiple-comparison test. Mean differences with an adjusted *P* value of ≤0.05 were deemed significant. ****, *P* ≤ 0.0001. ns, not significant.

Since the absence of *gmd* did not affect growth, we focused on strain Δ*gmd* for studying motility-related phenotypes. Strains lacking *gmd* had decreased motility in soft agar compared with WT bacteria (Fig. 1A). To further confirm the motility defect of strain Δ*gmd*, we replaced the *gmd* deletion locus with a wild-type copy of *gmd* (Fig. 1A). The motility phenotype of the revertant (strain Δ*gmd*::*gmd*) was identical to that of the WT. We also compared the motility phenotype of strain Δ*gmd* to that of a strain harboring premature stop codons that prevent translation of *gmd* (strain Δ*gmd*::gmd*^STOP^); the motility of this strain was similar to that of strain Δ*gmd* (Fig. 1A). These results show that loss of *gmd* limits motility, suggesting that absence of O-antigen alters swimming behavior.

To analyze the genetic interaction between *cdgD* and *gmd*, we generated a double mutant, strain Δ*cdgD* Δ*gmd*. This mutant had a motility phenotype intermediate between those of the single mutants (Fig. 1B). Next, we asked if the absence of *gmd* would also suppress the motility defect of a strain lacking the DGC CdgH. Earlier work showed that CdgH is an active DGC (26, 27). Similarly to what we observed in the Δ*cdgD* strain, the absence of *gmd* in the strain Δ*cdgH* background resulted in an intermediate motility phenotype (Fig. 1C). Collectively, these results show that the observed enhancements of motility in strains Δ*cdgD* and Δ*cdgH* are most likely mediated by bypass suppression in double mutants with Δ*gmd*.

### Lack of *cdgD*, *cdgH*, or *gmd* does not affect flagellum biogenesis.

**FIG 2:**
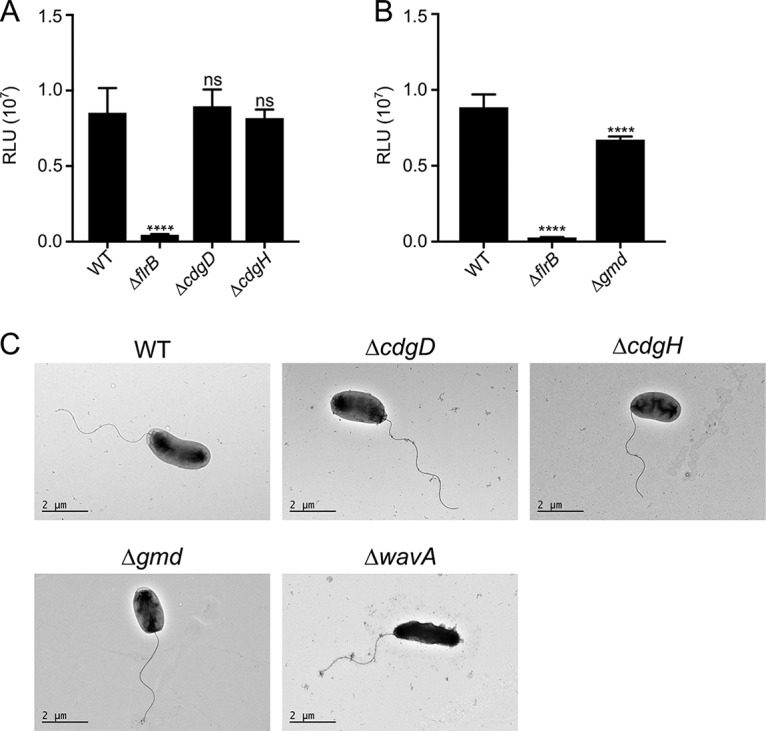
Flagellum biosynthesis is not affected in the absence of CdgD or CdgH and is decreased only slightly in the absence of Gmd. (A and B) Expression of the transcriptional fusion P*flaA*-*luxCADBE* in (A) WT, Δ*flrB*, Δ*cdgD*, and Δ*cdgH* strains and (B) WT, Δ*flrB*, and Δ*gmd* strains. The bar graphs represent the means and standard deviations of the relative light units (RLU) produced, which are directly proportional to *flaA* expression levels. Means were compared to the WT data using ANOVA and Dunnett's multiple-comparison test. Mean differences with an adjusted *P* value of ≤0.05 were deemed significant. ****, *P* ≤ 0.0001. ns, not significant. (C) Transmission electron microscopy of negatively stained WT, Δ*cdgD*, Δ*cdgH*, Δ*gmd*, and Δ*wavA* cells from exponentially growing cultures. Scale bars, 2 µm.

A potential mechanism for motility inhibition by CdgD and CdgH could be downregulation of flagellar biogenesis. To determine if the absence of the DGCs or Gmd affects the expression of *flaA*, the gene that encodes the major flagellin, we fused the regulatory region of *flaA* to the reporter *luxCADBE* in plasmid pBBRlux. As expected, expression of this transcriptional fusion (pFY1122) was abrogated in a genetic background that lacked the direct positive regulator FlrB (Fig. 2A). Expression of *flaA* was not affected by the absence of CdgD or CdgH but was modestly reduced (77% of WT) in the absence of Gmd (Fig. 2A and B). Analysis of flagellum production using electron microscopy (EM) revealed that strains Δ*cdgD* and Δ*cdgH* make a single polar flagellum indistinguishable from that of the WT bacteria. Similarly, Δ*gmd* and Δ*wavA* bacteria had intact flagellum, although some cells lacking *wavA* showed an irregular cell body shape (Fig. 2C). We found that 94% of cells from the WT strain, 95% of cells from the Δ*gmd* strain, 93% of cells from the Δ*cdgD* strain, and 94% of cells from the Δ*cdgH* strain were flagellated (Table S2). These experiments suggest that CdgD, CdgH, and Gmd regulate motility by means other than controlling flagellar biosynthesis.

10.1128/mBio.00670-19.8 TABLE S2 Quantification of flagellated cells from electron microscopy images. Download Table S2, PDF file, 0.02 MB. Copyright © 2019 Zamorano-Sánchez et al. 2019 Zamorano-Sánchez et al. This content is distributed under the terms of the Creative Commons Attribution 4.0 International license.

### Contribution of CdgD, CdgH, and Gmd to motility and initial surface attachment dynamics.

Traditional soft agar motility assays measure the rates of bacterial growth and expansion on soft agar plates. Such motility most likely depends on a number of factors, including swimming speed, surface interactions which include landing and leaving, and growth on surface. Earlier studies suggested that c-di-GMP can impact swim speed as well as flagellum reversal frequency (39–41). To analyze the behavior of individual cells, we developed a toolbox of new metrics for high-speed microscopy data that capture the diverse range of single-cell activities (Table 1). These metrics offer a precise breakdown of how a given mutation influences the different stages of the swimming-to-surface transition process for V. cholerae. In order for a cell to attach, grow, and expand on a surface, cells need to (i) swim, (ii) land on a surface, (iii) stay attached to the surface, and (iv) transition to a sessile state, possibly with suppression of flagellum function/activity (Table 1). Moreover, postlanding behaviors, such as production of c-di-GMP to enhance surface commitment, may also contribute to growth and expansion on the surface. Intuitively, higher swim speeds lead to increased rates of colony expansion on plate assays, but the dependence on the other two metrics is more complex. For example, both cells that strongly attach to the surface and cells that never attach to the surface negatively impact the effective rates of colony expansion on the surface. Similarly, changes in the time evolution of c-di-GMP production after landing may influence surface growth and expansion.

**TABLE 1:** List of metrics used for the analysis of the motile-to-sessile transition

Metric	Definition
Mean trajectory speed	Swimming speed before surface landing; calculated with averaged frame-to-frame speed (5-ms interval) for an entire trajectory
Landing probability (LP)	Probability of landing based on swimming distance; calculated using the number of landing events vs the contour length of swimming trajectories
Surface residence time (SRT)	Duration of cell attachment on surface; measured using a combination of radius of gyration and speed with various cutoff time thresholds
Quiescent intervals (QI)	Periods of little or no movement for surface-attached cells; measured using a combination of radius of gyration and speed with various cutoff time thresholds

**FIG 3:**
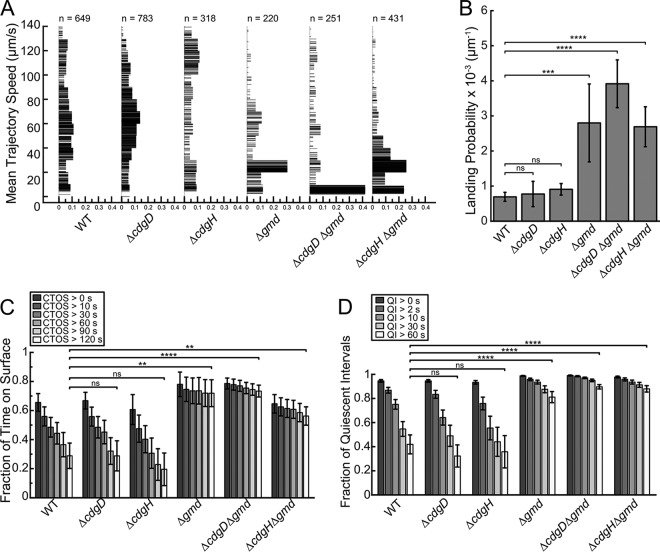
CdgD, CdgH, and Gmd affect surface motility at different stages of the motile to sessile transition. (A) Mean trajectory speeds for WT, Δ*cdgD*, Δ*cdgH*, Δ*gmd*, Δ*cdgD* Δ*gmd*, and Δ*cdgH* Δ*gmd* strains. The number of trajectories is indicated above the distribution plot for each strain. Each line in the plot represents one trajectory. The widths of the lines represent the distribution probabilities with a bin width of 10 µm/s. (B) Landing probabilities. (C) Fractions of cells with surface residence times greater than 0, 10, 30, 60, 90, and 120 s over the total time of presence for all swimming and surface-attached cells, for indicated strains. CTOS, continuous time on surface. (D) Fraction of cells with quiescent intervals of greater than 0, 2, 10, 30, and 60 s over the total time on surface for all surface-attached cells, for indicated strains. The standard error for each data set was estimated using 10,000 bootstrap random samplings with a sampling size that was 1.2× the number of trajectories or a value of 500, whichever was larger. Mean differences between the WT and the mutants with an adjusted *P* value of ≤0.05 were deemed significant. *, *P* ≤ 0.05; **, *P* ≤ 0.01; ***, *P* ≤ 0.001; ****, *P* ≤ 0.0001. ns, not significant.

The swimming-to-surface transition represents the earliest stage for the development of a biofilm. To elucidate how CdgD, CdgH, and Gmd modulate the swimming-to-surface transition, we compared the surface behaviors of single cells lacking *cdgD*, *cdgH*, or *gmd* and combinations of these mutations to the behavior of WT cells. The biofilm-forming abilities of these strains are further analyzed and discussed in subsequent sections. To analyze surface behaviors, we grew cells to stationary phase and the cells were imaged using a flow cell (in the absence of flow) with a high-speed camera, and the images were subsequently analyzed to extract various metrics using our toolbox. Measurements of near-surface motility trajectories of WT cells revealed a diverse distribution of swim speeds (Fig. 3A). Importantly, DGC mutations did not result in a general increase of swim speed for all cells, which would have been reflected in a uniform upward shift of the entire speed distribution. Rather, these mutations impacted different parts of this distribution differently. The Δ*cdgD* mutation resulted in a distribution rate for the 60-to-70-µm/s fraction that was slightly higher than that seen with the WT (Fig. 3A). The Δ*cdgH* mutation exhibited a pronounced shift of the speed distribution toward higher speeds, with one of the peaks corresponding to 110 to 120 µm/s (Fig. 3A). The swimming speed distribution of strain Δ*cdgD* versus strain Δ*cdgH* indicates the presence of different pathways for motility suppression.

The mutants that lacked *gmd* exhibited much larger populations of the slowest swimmers (for the 0-to-10, 10-to-20, and 20-to-30 μm/s fractions, the distributions were 11.8%, 10.5%, and 30.5%, respectively, for strain Δ*gmd* compared to 8.6%, 6.9%, and 5.2% for WT) at the expense of all other speeds (Fig. 3A). In fact, the absence of Gmd had a more dramatic impact on cell motility than loss of either DGC. Thus, the recovery of WT-like behavior in the Δ*cdgD* and Δ*cdgH* double mutants with the Δ*gmd* mutation stems from an increase in the subpopulation of the slowest swimmers rather than from a general decrease of swim speeds across the distribution.

We next measured the landing probability (LP), which is expressed as the number of landing events per micrometer of swimming distance for the entire cell population. The observed LP for WT was 0.69 ± 0.13 × 10^−3^ µm^−1^; the LPs for strain Δ*cdgD* and strain Δ*cdgH* were 0.77 ± 0.36 × 10^−3^ µm^−1^ and 0.91 ± 0.16 × 10^−3^ µm^−1^, respectively (Fig. 3B). These observations suggest that the absence of *cdgD* or *cdgH* does not affect the LP. On the other hand, the LP for strain Δ*gmd* was 2.80 ± 1.11 × 10^−3 ^µm^−1^, significantly higher than the LPs for the WT strain, strain Δ*cdgD*, and strain Δ*cdgH*. The double mutants Δ*cdgD* Δ*gmd* and Δ*cdgH* Δ*gmd* showed drastic increases in LP relative to the WT strain of 3.92 ± 0.68 × 10^−3^ µm^−1^ and 2.69 ± 0.57 × 10^−3^ µm^−1^, respectively.

We did not observe a significant difference in postlanding behaviors (metrics of surface residence time [SRT] and quiescent intervals [QI]; defined in Table 1) in strain Δ*cdgD* and strain Δ*cdgH* compared to the WT. However, strains lacking *gmd* had much higher fractions of cells that stayed on the surface compared to the WT strain and the single mutants Δ*cdgD* and Δ*cdgH* (Table 1). For example, the fraction of WT with a surface residence time of longer than 120 s was 0.29 ± 0.09, whereas the fraction for the Δ*gmd* strain was 0.72 ± 0.09, for the Δ*cdgD* Δ*gmd* strain was 0.73 ± 0.04, and for the Δ*cdgH* Δ*gmd* strain was 0.56 ± 0.06 (Fig. 3C). Similarly, cells lacking *gmd* remained quiescent for longer intervals than did the WT and single mutants Δ*cdgD* and Δ*cdgH*. For example, the fraction of WT cells that were quiescent for more than 60 s was 0.42 ± 0.08. In contrast, the fraction for the Δ*gmd* strain was 0.81 ± 0.05, for strain Δ*cdgD* Δ*gmd* was 0.90 ± 0.02, and for strain Δ*cdgH* Δ*gmd* was 0.88 ± 0.03 (Fig. 3D). Taking the data together, high-speed single-cell motility analyses revealed that DGCs (CdgD and CdgH) and Gmd impact different stages of the motility-to-biofilm lifestyle transition.

### Absence of CdgD or CdgH affects c-di-GMP accumulation.

**FIG 4:**
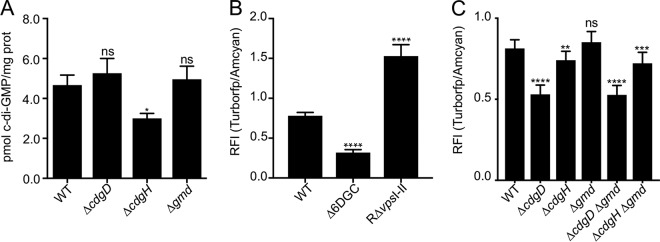
Absence of *cdgD*, *cdgH*, and *gmd* affected c-di-GMP accumulation in planktonic cultures and/or soft agar plates. (A) Bar graph of means and standard deviations of c-di-GMP concentrations (picomoles of c-di-GMP per milligram of protein) in exponentially growing cultures of WT, *ΔcdgD*, *ΔcdgH*, and *Δgmd* bacteria measured using HPLC-MS/MS. (B and C) Bar graph of means and standard deviations of c-di-GMP levels in (B) WT, *Δ*6DGC, and R*ΔvpsI-II* cells and (C) WT, *ΔcdgD*, *ΔcdgH*, Δ*gmd*, Δ*cdgD* Δ*gmd*, and Δ*cdgH Δgmd* cells. The accumulation of c-di-GMP was measured by analysis of reporter gene expression. The RFI represents the ratio between TurboRFP and AmCyan fluorescence intensities and is directly proportional to c-di-GMP levels. Means were compared to the WT data using ANOVA and Dunnett’s multiple-comparison test. Mean differences with an adjusted *P* value of ≤0.05 were deemed significant. *, *P* ≤ 0.05; **, *P* ≤ 0.01; ***, *P* ≤ 0.001; ****, *P* ≤ 0.0001. ns, not significant.

Cellular c-di-GMP levels modulate motility, the motility-to-biofilm transition, and biofilm formation; thus, we analyzed contributions of CdgD and CdgH to the cellular c-di-GMP pool. We first quantified the c-di-GMP pool in exponentially growing cells from planktonic cultures using high-performance liquid chromatography–tandem mass spectrometry (HPLC-MS/MS). We observed no significant changes in c-di-GMP levels in strain Δ*cdgD* compared to the WT cultures and a 36% reduction in c-di-GMP levels in strain Δ*cdgH* compared to the WT cultures, in agreement with a previous report (28) (Fig. 4A). Although Gmd impacts the motile-to-sessile transition (Fig. 1 and 3), in the absence of Gmd, there was no significant difference in intracellular c-di-GMP levels compared to that in the WT strain in exponentially growing planktonic cultures (Fig. 4A).

The motility phenotype of CdgD and CdgH was originally observed in cells growing in soft agar motility plates. We hypothesized that the roles of these DGCs and perhaps of Gmd in control of c-di-GMP homeostasis in cells growing in soft agar motility plates could be different from those in planktonic cultures. To measure c-di-GMP accumulation in living cells in solution culture, we adapted for use in V. cholerae a previously described c-di-GMP reporter (42). In this reporter, production of TurboRFP is regulated by three c-di-GMP riboswitches (Bc3 to Bc5) arrayed in tandem. Production of TurboRFP is repressed in the absence of c-di-GMP. The fluorescent protein AmCyan encoded in this biosensor is produced constitutively and is used as a normalizer. We validated this reporter in V. cholerae strains with low (*Δ*6DGC) (43) or high (RΔ*vps*I-II) (23, 25, 44) cellular c-di-GMP levels. The relative fluorescence intensity (RFI) of cells that express this reporter is directly proportional to c-di-GMP levels. Our results revealed that strain *Δ*6DGC had 43.5% of WT c-di-GMP levels whereas RΔ*vps*I-II had 195.6% of WT c-di-GMP levels in cells grown in soft agar (Fig. 4B). This result demonstrated that the c-di-GMP reporter allows detection of c-di-GMP levels below and above WT levels.

We next analyzed the abundance of c-di-GMP in Δ*cdgD*, Δ*cdgH*, and *Δgmd* cells grown on motility plates using the c-di-GMP reporter. The levels of c-di-GMP in the *ΔcdgD* strain were 65.1% of WT levels, whereas the levels of c-di-GMP in the *ΔcdgH* strain were 92.2% of WT levels. We did not observe a statistically significant difference in c-di-GMP accumulation in *Δgmd* cells compared to the WT (Fig. 4C). These results suggest that CdgD plays a more pronounced role than CdgH in c-di-GMP homeostasis in soft agar motility assays and that the lack of Gmd does not affect c-di-GMP accumulation. To determine if the absence of Gmd affects c-di-GMP accumulation in cells that lack either CdgD or CdgH, we measured c-di-GMP accumulation in the Δ*cdgD* Δ*gmd* and Δ*cdgH* Δ*gmd* double mutants grown in soft agar plates (Fig. 4C). These double mutants behaved like single Δ*cdgD* and Δ*cdgH* mutants, respectively. This further supports our finding that CdgD and CdgH contributed to c-di-GMP accumulation in motility plates and that Gmd did not have a noticeable effect on c-di-GMP accumulation under these conditions.

### CdgD, CdgH, and Gmd affect c-di-GMP accumulation during different stages of surface colonization.

**FIG 5:**
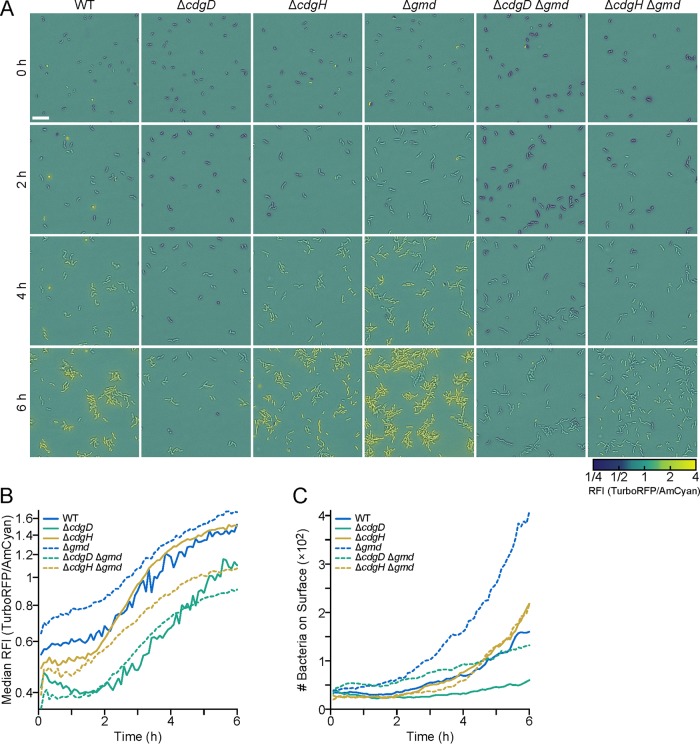
c-di-GMP levels were modulated by CdgD, CdgH, and Gmd in bacteria attached to flow cells. (A) Representative images of bacterial cells attached to the surface of a flow chamber. Images were taken 0, 2, 4, and 6 h after inoculation. The RFI represents the ratio between TurboRFP and AmCyan fluorescence intensities and is directly proportional to c-di-GMP levels. RFI values are represented by false coloring (according to the color bar scale) overlaid on bright-field images. Scale bar, 10 μm. (B) Plots of the median RFI in images of WT, Δ*cdgD*, Δ*cdgH*, Δ*gmd*, Δ*cdgD* Δ*gmd*, and Δ*cdgH* Δ*gmd* cultures for the first 6 h after inoculation in the flow cell. (C) Plots of the number of bacteria per fluorescence image over the 6-h time course.

Next, using the c-di-GMP reporter, we analyzed how c-di-GMP production changes during the initial stages of biofilm formation and how CdgD, CdgH, and Gmd contribute to this process. Representative images of cells expressing the c-di-GMP reporter at different time points are shown in Fig. 5A. The evolution of c-di-GMP accumulation is sigmoidal with three regimes: an initial lag period of various durations where there is no significant increase of c-di-GMP concentration after the cell lands on a surface, a period of rapid c-di-GMP increase, and a saturation period (Fig. 5B). Interestingly, *ΔcdgD* cells, which swim at speeds similar to WT cell speeds, had markedly lower levels of c-di-GMP than WT cells during all three stages; on average, throughout the 6-h time course evaluated, strain *ΔcdgD* had 66% of WT levels. In contrast, the accumulation of c-di-GMP in *ΔcdgH* cells was quite similar to that seen in WT cells. During the first 2 h, strain *ΔcdgH* had 88% of WT levels, but for the remainder of the tracked 6-h period, c-di-GMP levels were not significantly different from those in WT cells. The *Δgmd* mutant cells had higher levels of c-di-GMP than the WT cells: During the first 2 h, strain *Δgmd* had 26% higher levels than the WT strain, and on average throughout the 6 h, these cells had 20% higher levels than the WT cells (Fig. 5B). These observations suggest that, in contrast to how mutations alter prelanding swim speed modulation behavior, *ΔcdgD* exerts a stronger influence than *ΔcdgH* in terms of c-di-GMP accumulation in cells on the surface. The absence of Gmd, which has strong consequences for swimming motility, resulted in an increase in c-di-GMP levels compared to the WT levels seen postattachment.

Given that CdgD, CdgH, and Gmd modulate intracellular c-di-GMP levels and motility in V. cholerae differently, we evaluated the intracellular c-di-GMP levels in double mutants. Results obtained from tracked cells on the surface during the first 6 h suggest that the effects of modulation of c-di-GMP levels in the absence of CdgD, CdgH, and Gmd are not additive. The evolution of c-di-GMP concentrations over time for *ΔcdgD Δgmd* double mutants was comparable to the results seen with the *ΔcdgD* single mutants. However, the *ΔcdgH Δgmd* double mutant had much lower c-di-GMP levels than the WT cells, even though the *ΔcdgH* and *Δgmd* single mutants had c-di-GMP levels that were similar to or increased over those seen with the WT strain (Fig. 5B).

All the genetic backgrounds analyzed had similar numbers of cells on the surface at the beginning of our 6-h recording (Fig. 5C). On average throughout the 6 h, there were 40% fewer *ΔcdgD* cells and 17% more *ΔcdgH* cells than WT cells on the surface. We found that there were approximately double the numbers of *Δgmd* cells that adhered to the surface compared to the WT over the duration of the experiment. The number of *ΔcdgD Δgmd* cells was 45% higher than the number of WT cells until ∼4.5 h, but the number of *ΔcdgD Δgmd* cells that adhered to the surface at 6 h was 5% lower than the number of WT cells. On the basis of these observations, it appears that the *ΔcdgD Δgmd* double mutant has an intermediate surface colonization phenotype compared to the phenotype of the *ΔcdgD* and *Δgmd* single mutants. The number of *ΔcdgH Δgmd* cells that adhered was similar to the number seen with strain *ΔcdgH* and ∼50% lower than the number seen with strain *Δgmd*. These results suggest that the lack of Gmd results in an increase in the number of surface-attached cells compared to the WT only when both CdgD and CdgH are present.

### The absence of Gmd promotes biofilm formation.

**FIG 6:**
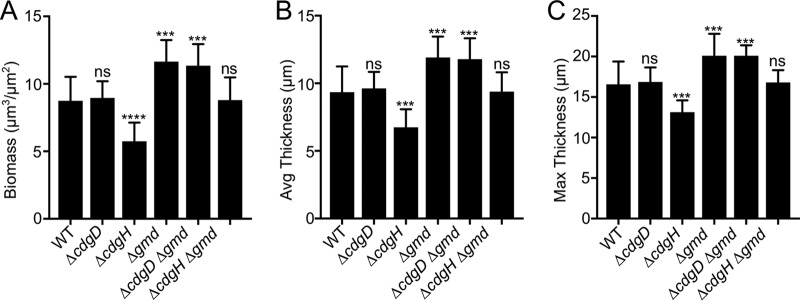
Deletion of *gmd* promotes biofilm formation. (A to C) Bar graphs of means and standard deviations of the (A) biomass, (B) average thickness, and (C) maximum thickness of biofilms grown for 24 h in a flow cell system. Four biological replicates were analyzed. Images were obtained using confocal laser scanning microscopy and were analyzed with COMSTAT 2.0 software. Means were compared to WT data using ANOVA and Dunnett’s multiple-comparison test. Mean differences with an adjusted *P* value of ≤0.05 were deemed significant. ***, *P* ≤ 0.001; ****, *P* ≤ 0.0001.

We next analyzed biofilm formation in the Δ*cdgD*, Δ*cdgH*, Δ*gmd*, Δ*cdgD* Δ*gmd*, and Δ*cdgH* Δ*gmd* mutants. Biofilms were grown under constant flow conditions for 24 h. We used COMSTAT 2 biofilm image analysis software (45) to determine the biomass, average thickness, and maximum thickness of the mature biofilms. Data are plotted in Fig. 6. Biofilms of the Δ*cdgD* strain appeared similar to WT biofilms; however, biofilms formed by strain *ΔcdgH* had lower biomass and thickness. The delay in surface colonization by the Δ*cdgD* strain relative to the WT strain (Fig. 5C) and the reduction in biofilm biomass in the Δ*cdgH* strain (Fig. 6) were not unexpected as strains with low concentrations of c-di-GMP often have either delayed or decreased biofilm-forming abilities (25).

The *Δgmd* strain formed biofilms that were higher in biomass and thickness than the WT biofilms. The Δ*cdgD Δgmd* double mutant behaved similarly to the *Δgmd* single mutant. This observation suggests that CdgD is not required for the increased biofilm formation observed in strain *Δgmd*. The Δ*cdgH Δgmd* double mutant behaved similarly to the WT strain. Considering that strain Δ*cdgH* formed thinner biofilms than the WT strain, this result suggests that the absence of Gmd can stimulate biofilm formation in the absence of CdgH but not to its maximum capability.

## DISCUSSION

### Identification of strong motility regulators.

At present there is not a detailed understanding of how the different c-di-GMP modulators in V. cholerae differentially influence motility. Moreover, given the large number of these modulators, it is not clear how effector strengths compare or how they collectively influence motility in the cell. The intrinsic difficulty of addressing these issues is compounded by the contingent difficulty of measuring outcomes with cellular resolution and with strong statistics. In this study, we analyzed how DGCs CdgD and CdgH control motility. We also developed new tools to assess motility behavior and determined how outputs of new analysis tools can be related to traditional readouts such as those from bulk agar plate assays.

We first performed transposon mutagenesis to identify extragenic suppressors of increased motility in the Δ*cdgD* deletion mutant. Our initial screen identified suppressor mutants with wild-type motility. Most of the suppressor mutants that we identified had sustained insertions into genes involved in the biosynthesis of LPS, in particular, in O-antigen biosynthesis. The inspection of the motility phenotype of a mutant deficient in O-antigen biosynthesis (strain *Δgmd*) by itself or together with a null mutation of *cdgD* or *cdgH* clearly showed that O-antigen production impacts V. cholerae motility. These observations suggest that the absence of O-antigen functions as a bypass suppressor of hypermotility in strain Δ*cdgD* and strain Δ*cdgH*, and the corresponding genes are most likely not in the same regulatory pathway. We found that expression of *flaA*, which encodes the major flagellin, was not affected in Δ*cdgD* or Δ*cdgH* mutants and was decreased only modestly in the Δ*gmd* strain. While we could not reliably measure flagellum length using electron microscopy, we did not observe any differences in the numbers of flagellated cells. Furthermore, the morphologies of the flagella made by the WT strain, strain Δ*cdgD*, strain Δ*cdgH*, and strain Δ*gmd* were similar, suggesting that flagellum biosynthesis or assembly is not affected in these genetic backgrounds and that motility inhibition is controlled at a posttranslational level.

### Differential modulation of swimming motility by DGCs.

There are well-characterized mechanisms that involve c-di-GMP-mediated inhibition of flagellar rotation. In Escherichia coli, changes in the global c-di-GMP pool regulate motility through YcgR, a protein that acts as a motor brake (39–41, 46, 47). In Bacillus subtilis, c-di-GMP inhibits swarming through the receptor DgrA/MotI by acting as a clutch (48–50). In these systems, c-di-GMP modules regulate motility reversibly, through protein-protein interactions. We found that deletion of *cdgH* in V. cholerae led to a systematic shift toward higher swim speeds than were observed for WT cells. Posttranscriptional regulation of flagellar motility in V. cholerae has not been explored in great detail. V. cholerae lacks orthologues of YcgR and MotI but makes proteins with PilZ domains that bind c-di-GMP and regulate motility (15, 51, 52). It is yet to be determined whether V. cholerae Plz proteins regulate motor and stator interactions in response to changes in c-di-GMP levels.

### Landing on a surface, staying on a surface.

We used landing probability metrics to quantify the surface interaction tendency of cells. To date, we have analyzed only the initial landing events that can be linearly fitted (Fig. 3B; see also Fig. S5 in the supplemental material). We did not observe significant differences among the initial LPs for the WT strain, strain Δ*cdgD*, and strain Δ*cdgH*; however, the overall landing profile for strain Δ*cdgD* seems to be different from those of WT and strain Δ*cdgH* (Fig. S5). We found that strain Δ*gmd* had higher landing probability; the LPs of strain Δ*gmd*, strain Δ*cdgD* Δ*gmd*, and strain Δ*cdgH* Δ*gmd* were 305%, 468%, and 290% higher than the LP of the WT strain, respectively. The regulation of landing behavior of V. cholerae appears to be complex and multifactorial, and the details of the mechanisms governing how CdgH, CdgD, and Gmd influence landing probability are not clear at present.

10.1128/mBio.00670-19.6 FIG S5 Landing probability was calculated using the initial linear period. The cumulative contour lengths determined for all swimming cells are plotted against the cumulative landing events (open circles). The black line in each subplot indicates the initial linear portion that was used to obtain the averaged swimming distance per landing, which represents the inverse of the landing probability value. Download FIG S5, TIF file, 33.1 MB. Copyright © 2019 Zamorano-Sánchez et al. 2019 Zamorano-Sánchez et al. This content is distributed under the terms of the Creative Commons Attribution 4.0 International license.

The current paradigm assumes that V. cholerae cells move very little after they land on a surface. However, our high-speed microscopy observations revealed a rich diversity of single-cell movements after landing (e.g., spinning, vibrating, reentrant near-surface swimming) that were presumably due to flagellar rotation (the time scale for pilus activity is much longer than that for flagellar activity). The absence of either *cdgD* or *cdgH* had no impact on surface residence time or quiescent interval. The strong observed correlation between longer surface residence time and longer quiescent interval for the three strains lacking *gmd* suggests that longer observed periods of postlanding motility suppression may be the key to staying on the surface longer and that the absence of O-antigen in the *gmd* mutation (or its downstream consequences) may favor such suppression of surface movements.

### Differential modulation of c-di-GMP production by DGCs of surface-attached cells.

The lack of CdgD, which had minimal influence on prelanding swim speeds compared to lack of CdgH, resulted in markedly lower levels of c-di-GMP than were observed in WT cells for the first 6 h after landing (the duration of the experiment). In contrast, the behavior of the *ΔcdgH* mutant was similar to that of WT. This implies that there is some division of labor between CdgD and CdgH, with the latter having more effect on prelanding swim speed and the former enforcing the production of c-di-GMP levels postlanding.

We expected to observe the simultaneous coregulation of c-di-GMP levels and motility by multiple proteins in V. cholerae. Our results revealed that both c-di-GMP production and the tendency for biofilm formation were increased in the Δ*gmd* mutant, which showed a strong inhibition of motility (in fact, an even stronger motility impact than lack of DGCs). As a rough test of what happens during comodulation of c-di-GMP, we examined the behavior of double mutants. Analyses of the *ΔcdgH Δgmd* double mutant suggested that modulation of c-di-GMP levels by different effectors can be negatively cooperative rather than simply additive as the double mutant had much lower c-di-GMP levels than the WT strain, even though the *ΔcdgH* and *Δgmd* single mutants had c-di-GMP levels that were similar to or increased over those seen with the WT.

### Role of LPS in motility and biofilm formation.

We found that the Δ*gmd* strain had an increased capacity to deposit biomass on the surface relative to the WT strain. LPS production and O-antigen production have been shown to regulate bacterial motility and biofilm formation by diverse mechanisms. For instance, a deficiency in O-antigen ligation due to a mutation in *waaL* affects the ability of Vibrio fischeri to spread in soft agar plates (53). It was also shown that V. fischeri sheds LPS (54), but it is unclear what effect LPS shedding has on motility behavior. In E. coli, mutations that affect the proper assembly of the LPS core result in loss of flagella and promote biofilm formation (55, 56). It has been shown that defects in the structure of the LPS core in E. coli are sensed through the Rcs phosphorelay and control flagellar gene expression (46). The mechanism by which the Rcs phosphorelay regulates flagellar biosynthesis in response to cell envelope stress in a close relative of E. coli, namely, Salmonella enterica serovar Typhimurium, has been recently reported (57). In response to cell membrane perturbations, the Rcs phosphorelay promotes production of a protein with a degenerate EAL motif, RflP, which targets the flagellar gene regulator FlhD_4_C_2_ for degradation by ClpXP. Interestingly, RflP does not have phosphodiesterase activity and also does not bind c-di-GMP; therefore, it is unclear if c-di-GMP is involved in this process (58).

Although the biofilm communities formed by WT and *Δgmd* strains may be different, our results showed that lack of O-antigen biosynthesis results in increased surface biomass accumulation and increased c-di-GMP levels in V. cholerae. The *Δgmd* mutant deficient in perosamine biosynthesis that we used for this study autoaggregates in liquid culture. Given that the *ΔwavA* mutant, which lacks O-antigen and a heptose in a core LPS, does not aggregate, autoaggregation is likely to be a consequence of cell-cell interactions through glycans in the LPS core (59). We further speculate that these interactions promote surface attachment and perhaps signal c-di-GMP accumulation. Future work will aim to identify signal transduction cascades that link O-antigen, surface sensing, and c-di-GMP in V. cholerae.

The levels of c-di-GMP affect the synthesis and/or presence of several components of the V. cholerae biofilm matrix (i.e., exopolysaccharides, matrix proteins, and adhesion proteins). It appears that c-di-GMP metabolizing enzymes contribute to different stages of biofilm progression. Our results suggest that a circuit controlled by the activity of CdgD and unknown receptors specifically affects the initial stages of biofilm development; this is in agreement with previous work performed by our group that showed that the absence of CdgD had a pronounced effect at early stages of biofilm formation but almost no effect on formation of mature biofilms (23). Additionally, CdgD is not a regulator of *Vibrio* exopolysaccharide (VPS) biosynthesis, in particular, of *vpsL* expression (23, 25). In contrast, CdgH is required for optimal VPS production and strains lacking CdgH have a reduced capacity to form biofilms (23, 28). Prior studies have documented that VPS impacts swimming behavior in motility plates (17). However, it is yet to be determined if the absence of VPS affects the distribution of swimming speeds and whether decreased VPS production in strain *ΔcdgH* is partly responsible for the motility phenotypes of CdgH. Identification of the effectors that control the type of motility behaviors that we uncovered using single-cell tracking and high-speed microscopy will allow us to propose models that allocate specific c-di-GMP signaling components within phenotype-specific regulatory circuits.

The data reported here suggest that there is a division of labor between different DGCs; CdgH has a marked impact on prelanding swim speeds, whereas CdgD contributes strongly to the postlanding c-di-GMP increase that initiates surface sensing and transduction. Moreover, modulation of c-di-GMP levels by different motility effectors (such as DGCs and LPS genes) is not additive but rather shows some level of cooperativity.

## MATERIALS AND METHODS

### Strains and growth conditions.

The bacterial strains and plasmids used in this study are listed in Table S3 in the supplemental material. V. cholerae and Escherichia coli strains were grown in lysogeny broth (LB) (1% tryptone, 0.5% yeast extract, 1% NaCl, pH 7.5) at 30°C and 37°C, respectively. Antibiotics were added in the following concentrations: ampicillin, 100 µg/ml in LB cultures and LB agar; kanamycin, 35 µg/ml in LB cultures and 50 µg/ml on LB agar; gentamicin, 10 µg/ml in LB cultures and 15 µg/ml on LB agar. LB agar and LB soft agar plates were prepared with 1.5% agar and 0.3% agar, respectively.

10.1128/mBio.00670-19.9 TABLE S3 Bacterial strains and plasmids used in this study. Download Table S3, PDF file, 0.04 MB. Copyright © 2019 Zamorano-Sánchez et al. 2019 Zamorano-Sánchez et al. This content is distributed under the terms of the Creative Commons Attribution 4.0 International license.

### Transposon mutagenesis and motility screen.

The suicide plasmid pSC189 containing a *mariner*-based transposon with a kanamycin resistance cassette was used to conduct random transposon-insertion mutagenesis. Selection for transposon insertion was performed on LB agar plates with 50 µg/ml kanamycin. Transposon mutant libraries were grown overnight in 96-well plates and transferred to LB soft agar plates (150 by 15 mm) using a 96-pin plate replicator. Plates were incubated for 7 h at 30°C, and mutants with motility comparable to that of the WT were selected for a secondary screen. The growth of selected mutants was compared to the WT growth; mutants affected in growth were discarded. Mutants that recapitulated the motility phenotype in smaller-scale plate motility assays were selected for transposon insertion mapping.

### Transposon insertion mapping.

Transposon insertions were mapped by PCR with the primers described in Table S4 and were synthesized by Integrated DNA Technologies. The initial round of arbitrary amplification was performed with primers Arb1 and seq5. The last round of amplification consisted of a nested PCR with secondary transposon-specific primer seq3 and primer Arb4. The nested PCR products were run in 1% agarose gels and purified using a PCR cleanup gel extraction kit (Macherey-Nagel). Primer seq3 was used for Sanger sequencing.

10.1128/mBio.00670-19.10 TABLE S4 Primers used in this work. Download Table S4, PDF file, 0.02 MB. Copyright © 2019 Zamorano-Sánchez et al. 2019 Zamorano-Sánchez et al. This content is distributed under the terms of the Creative Commons Attribution 4.0 International license.

### Recombinant DNA techniques.

All recombinant techniques were performed using standard procedures. All primers used in this study are shown in Table S4 and were synthesized by Integrated DNA Technologies. A description of the generation of genetic constructs is detailed in Text S1 in the supplemental material.

10.1128/mBio.00670-19.1 TEXT S1 Extended methods. Download Text S1, DOCX file, 0.02 MB. Copyright © 2019 Zamorano-Sánchez et al. 2019 Zamorano-Sánchez et al. This content is distributed under the terms of the Creative Commons Attribution 4.0 International license.

### Generation of in-frame deletion mutants and *gfp*-tagged strains.

Generation of deletions by double recombination was performed as previously described (12, 60). V. cholerae strains were tagged with the green fluorescent protein gene (*gfp*) as previously described (11). The *gfp*-tagged V. cholerae strains were verified by PCR.

### Soft agar motility assay.

Soft agar motility assays were carried out in 150-mm-by-15-mm petri dishes filled with 100 ml of LB containing 0.3% agar. Motility plates were dried for at least 4 h before stabbing of individual colonies and incubating at 30°C for 16 h (Fig. 1A), 14 h (Fig. 1B), and 13 h (Fig. 1C). Experiments were done in triplicate. The diameters of migration from at least nine independent colonies were averaged and subjected to statistical analysis using GraphPad Prism 7.

### Luminescence assay.

Overnight cultures of V. cholerae cells grown in LB were diluted 1:1,000 in LB containing 5 µg/ml chloramphenicol. Cultures were grown at 30°C and harvested at mid-exponential phase (optical density at 600 nm [OD_600_] of 0.3 to 0.4) for luminescence reading. Luminescence was measured using a Perkin Elmer Victor3 multilabel counter and is reported in counts per minute per milliliter divided by OD_600_. Assays were done with three independent biological replicates. Four technical replicates were measured for all assays. Statistical analysis was performed using GraphPad Prism 7.

### Transmission electron microscopy and quantification of flagellated cells.

Transmission electron microscopy (TEM) analyses (Fig. 2C) were performed on exponentially growing cells as described previously (14). For quantification of flagellated cells, we made modifications aimed to minimize perturbations that could cause flagellum detachment. Bacteria were diluted in phosphate-buffered saline (PBS) to yield an OD_600_ of 0.2 to 0.3. Each TEM sample was prepared by floating a 300-mesh Formvar carbon film grid (Electron Microscopy Sciences, Hatfield, PA) on a 100-µl drop of each diluted culture. After 2 min, each grid was washed gently by touching it to a drop of PBS and was negatively stained with 1% (wt/vol) aqueous uranyl acetate solution for 90 s. After removal of uranyl acetate, the grids were air-dried for 10 min and examined using a JEOL JEM-1400 transmission microscope. Bacterial cells in representative TEM images were used to quantify flagellated cells. Flagellated cells and nonflagellated cells were marked with green dots and yellow dots, respectively. The dot markers were counted in Adobe Illustrator CS6. Clustered cells and daughter cells of dividing cells were not included in the evaluation.

### Single-cell analysis of near-surface motility behaviors.

The near-surface motility behaviors were studied using high-speed microscopy and tracking as described previously (10) with minor modifications. Briefly, cells were grown in LB at 30°C for ∼18 or ∼22 h and then diluted 1:1,000 with 2% LB (0.02% tryptone, 0.01% yeast extract, 1% NaCl, pH 7.5). The diluted cells were injected into sticky-Slide VI 0.4 flow cells (ibidi GmbH) and imaged using an Olympus IX83 inverted microscope with a 100× oil objective. Images were captured using a Phantom V12.1 high-speed camera (Vision Research) at 200 frames per s. Imaging typically lasted 5 to 10 min after injection. Data processing was performed using MATLAB (MathWorks) with an algorithm developed in-house (see Text S1 in the supplemental material for more details). Metrics used for characterization of the swimming-to-surface attachment process of the cells are listed in Table S3.

### Flow cell biofilm studies using confocal laser scanning microscopy.

Analyses of biofilm formation in flow cell chambers were performed as previously reported (14). Quantitative analysis of biomass, average thickness, and maximum thickness was carried out with COMSTAT 2 software (45).

### Quantification of c-di-GMP using HPLC-MS/MS.

To quantify c-di-GMP concentrations, we extracted nucleotides as previously reported (14). All samples and standards were analyzed by HPLC-MS/MS with previously reported instrumentation and parameters (14). An aliquot of 5 ml of culture was used to determine the protein concentration with a bicinchoninic acid assay (Thermo Fisher Scientific). The concentration of c-di-GMP in each sample is expressed as picomoles of c-di-GMP per milligram of total protein. Quantification was performed on three biological replicates.

### Quantification of c-di-GMP relative abundances in motility plates using a biosensor.

Cells containing the c-di-GMP biosensor (pFY4535) were transferred to motility plates as described above. After 16 h of growth at 30°C, plates were imaged in three channels using a Bio-Rad ChemiDoc MP imaging system. White Epi illumination with no filters was used to image cell migration; blue Epi illumination and a 530/28 filter were used to detect fluorescence from AmCyan; green Epi illumination and a 605/50 filter were used to detect fluorescence from TurboRFP. The exposure time was adjusted for each channel to avoid saturation. The mean fluorescence intensity of AmCyan and TurboRFP near the site of inoculation was quantified using the “Volume” tools of Image Lab 4.0.1 software. A circle was draw over the pixels with the highest intensity (close to the site of inoculation). The numbers of pixels contained in the circle were the same for each sample. The RFI was calculated from the ratios of the mean fluorescence intensities of the TurboRFP and the AmCyan channels. Three independent experiments were performed; 17 independent motility halos were analyzed in total.

### Quantification of single-cell c-di-GMP relative abundances in flow cells using a biosensor.

Cells containing the c-di-GMP biosensor were streaked onto an LB agar plate containing 20 μg/ml gentamicin and incubated overnight at 30°C. A single colony from the plate was inoculated into LB containing 15 μg/ml gentamicin and was incubated for ∼18 h at 30°C with shaking at 200 rpm. This overnight culture was diluted 1:100 into 2% LB (0.02% tryptone, 0.01% yeast extract, 1% NaCl, pH 7.5) and inoculated into the flow cell. Flow cells were prepared and inoculated as previously described (61) with the following modifications. The flow cell was purchased from ibidi (sticky-Slide VI^0.4^ with a glass coverslip), and an in-line injection port (ibidi) was used at the inlet.

Images were taken using an Andor Neo scientific complementary metal-oxide-semiconductor (sCMOS) camera with IQ software on an Olympus IX83 microscope equipped with a 100× oil objective, a 2× multiplier lens, and a Zero Drift Correction autofocus system. Bright-field images were taken every 5 s (30 ms of exposure time). Two fluorescence images were taken every 5 min (100 ms of exposure time) using a Lambda LS (Sutter Instrument) xenon arc lamp and a red fluorescent protein (RFP) (for TurboRFP) or cyan fluorescent protein (CFP) (for AmCyan) filter. Total recording time was ∼10 h, resulting in ∼7,200 bright-field images and ∼240 fluorescence images (2 by 120). The image size was 67 by 67 µm^2^ (2,048 by 2,048 pixels).

The image analysis algorithms and software were adapted from methods that were previously described (61) and were written in MATLAB R2015a (MathWorks). The function “regionprops.m” was used to calculate the number of bacteria in each image. The RFI values were calculated by dividing the intensity value of TurboRFP by that of AmCyan.
