# Correction for Zamorano-Sánchez et al., “Functional Specialization in Vibrio cholerae Diguanylate Cyclases: Distinct Modes of Motility Suppression and c-di-GMP Production”

**DOI:** 10.1128/mBio.01960-20

**Published:** 2020-09-22

**Authors:** David Zamorano-Sánchez, Wujing Xian, Calvin K. Lee, Mauro Salinas, Wiriya Thongsomboon, Lynette Cegelski, Gerard C. L. Wong, Fitnat H. Yildiz

**Affiliations:** a Department of Microbiology and Environmental Toxicology, University of California, Santa Cruz, Santa Cruz, California, USA; b Department of Bioengineering, University of California, Los Angeles, Los Angeles, California, USA; c Department of Chemistry and Biochemistry, University of California, Los Angeles, Los Angeles, California, USA; d California Nano Systems Institute, University of California, Los Angeles, Los Angeles, California, USA; e Department of Chemistry, Stanford University, Stanford, California, USA

## AUTHOR CORRECTION

Volume 10, no. 2, e00670-19, 2019. https://doi.org/10.1128/mBio.00670-19. On PDF page 7, the last line from the last paragraph reads “by three c-di-GMP riboswitches (Bc3 to Bc5) arrayed in tandem.” It should read “by two c-di-GMP riboswitches from the Bc3 to Bc5 array found in tandem in the genome of Bacillus thuringiensis.” In the Text S1 file, page 4, paragraph two, the statement “The assembled product (pFY4357) contains all the elements of the original biosensor, two strong promoters (Pbe region) driving expression of the genes encoding AmCyan and TurboRFP, and three c-di-GMP riboswitches Bc3, Bc4 and Bc5, that modulate production of TurboRFP” should read “The assembled product (pFY4357) contains two strong promoters (Pbe region) driving expression of the genes encoding AmCyan and TurboRFP, and two c-di-GMP riboswitches Bc3 and Bc4 that modulate production of TurboRFP.”

These corrections do not change the conclusions of the manuscript.

