# Supplementary figures and images for: Functional Specialization in Vibrio cholerae Diguanylate Cyclases: Distinct Modes of Motility Suppression and c-di-GMP Production
Source: mBio. 2019 Apr 23;10(2):e00670-19. doi: 10.1128/mBio.00670-19 (PMC6479008; doi:10.1128/mBio.00670-19)

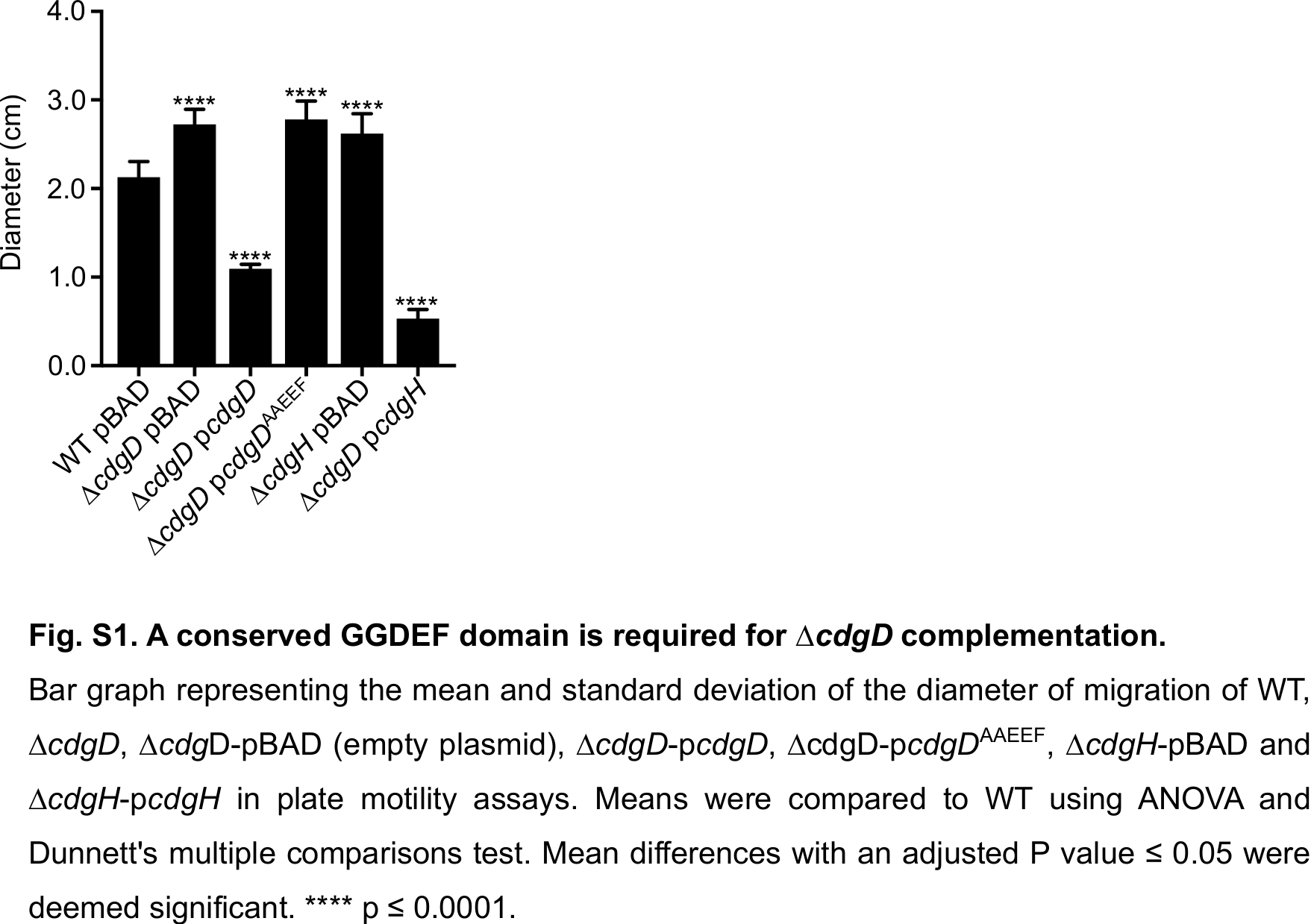

Supplement: FIG S1 [file mBio.00670-19-sf001.tif]

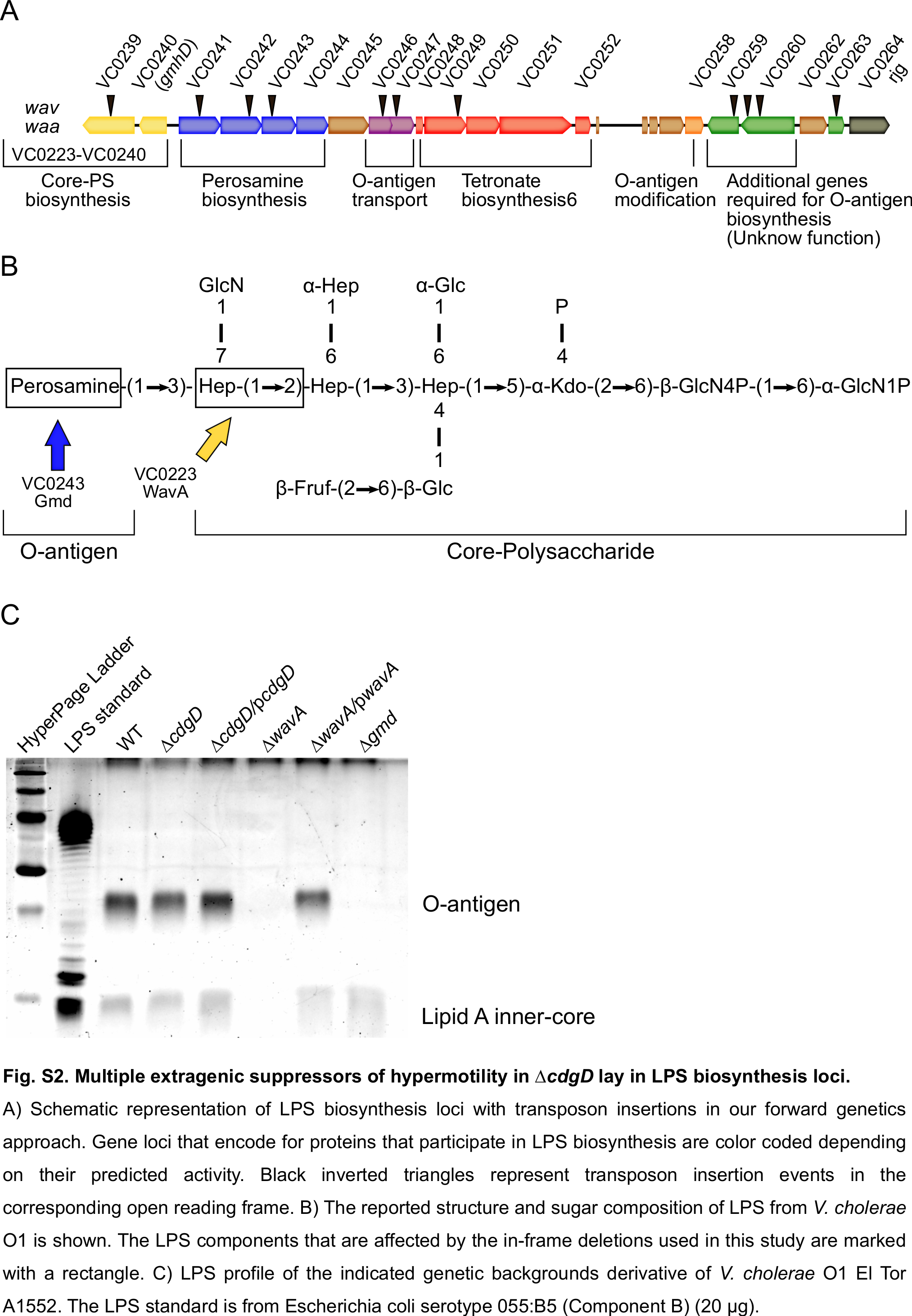

Supplement: FIG S2 [file mBio.00670-19-sf002.tif]

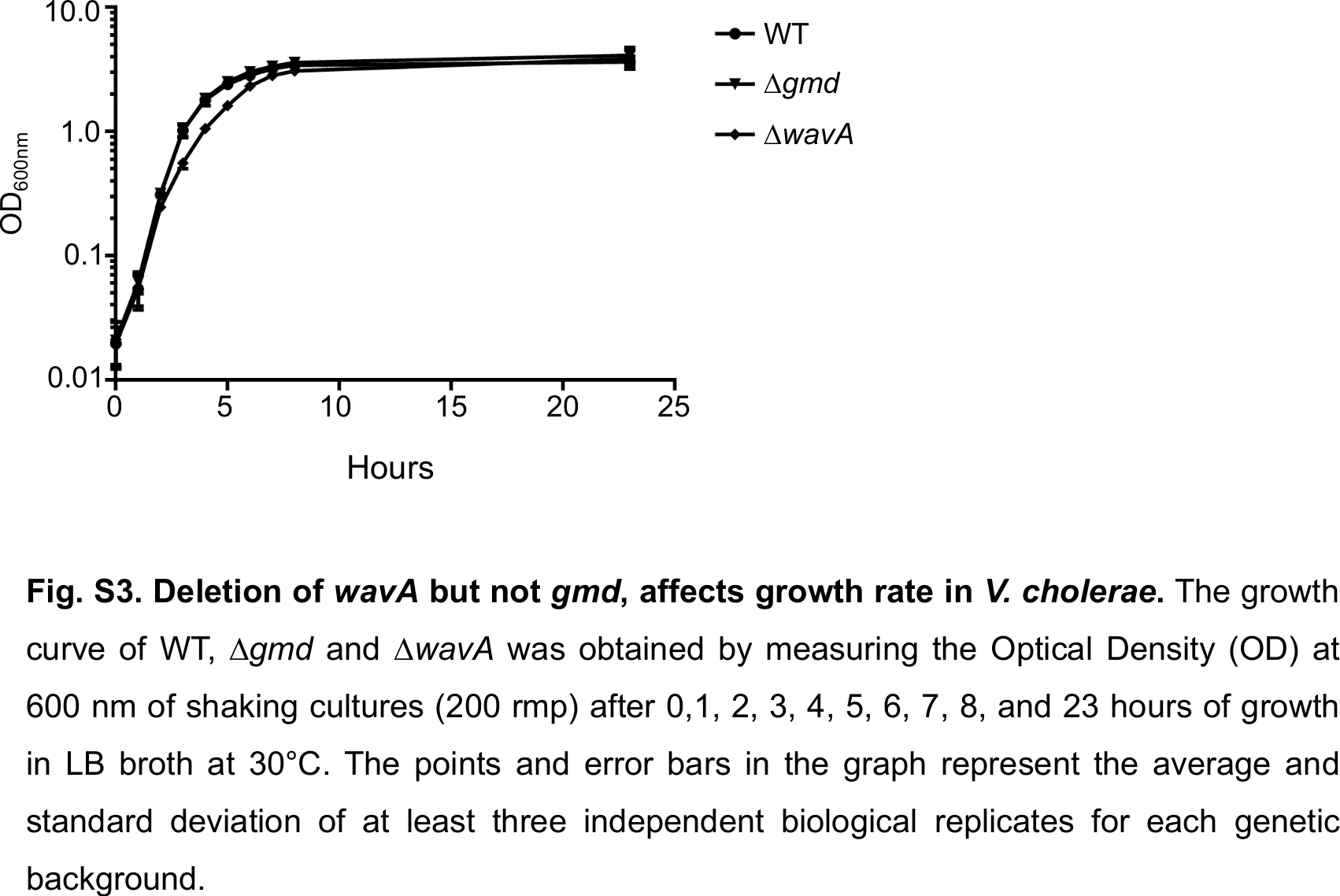

Supplement: FIG S3 [file mBio.00670-19-sf003.tif]

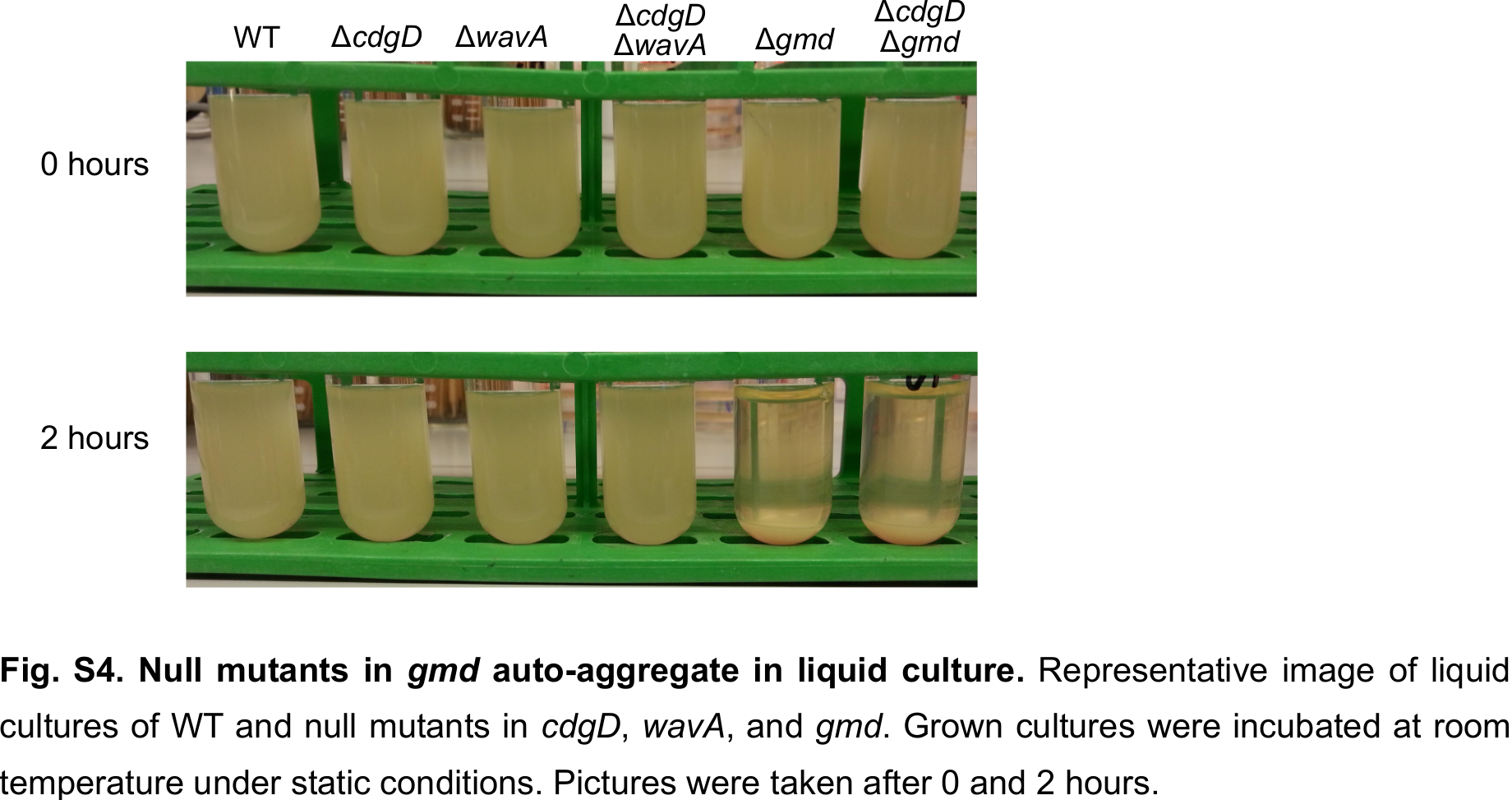

Supplement: FIG S4 [file mBio.00670-19-sf004.tif]

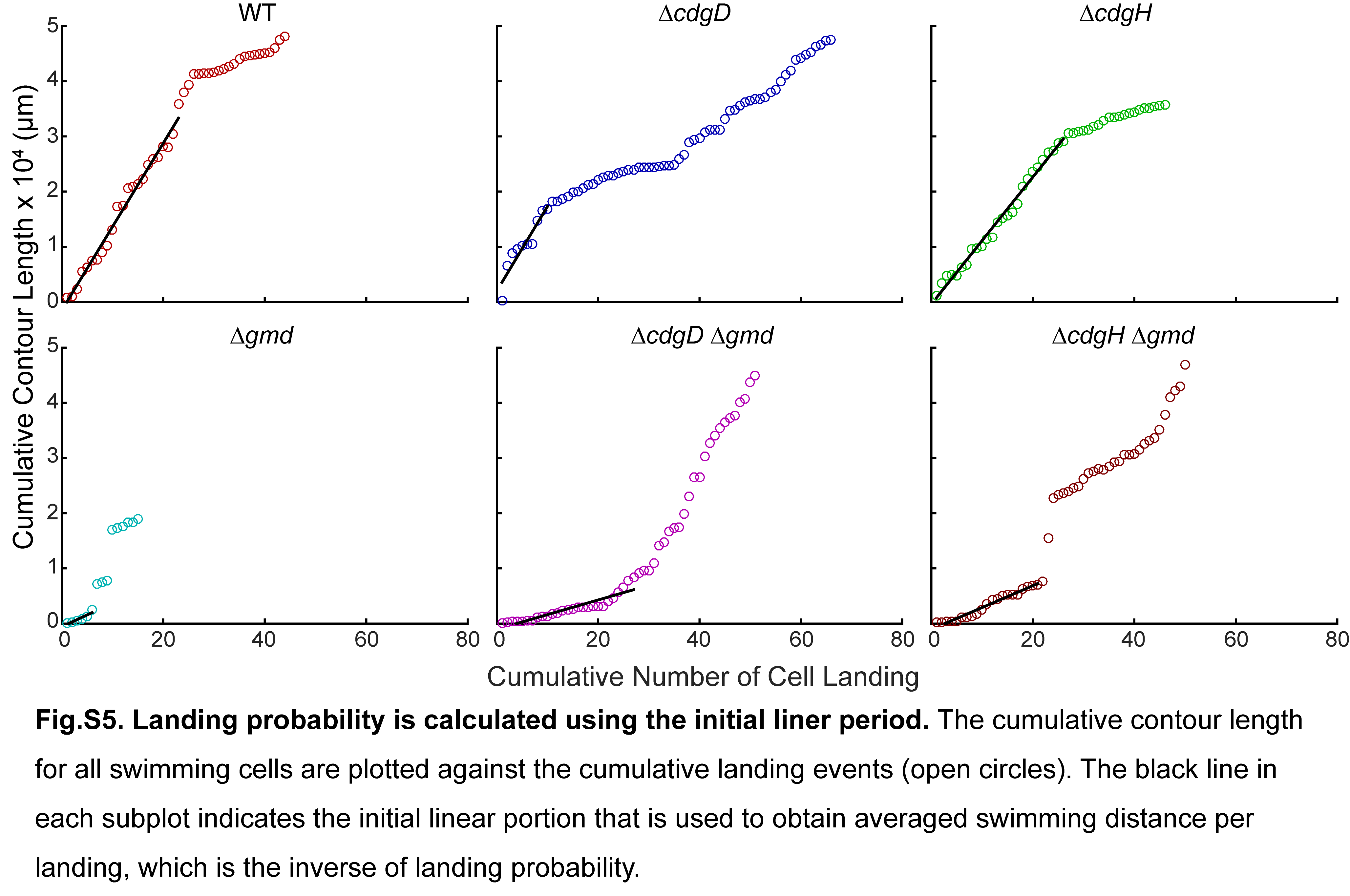

Supplement: FIG S5 [file mBio.00670-19-sf005.tif]
