# Supplementary material for: Functional Specialization in Vibrio cholerae Diguanylate Cyclases: Distinct Modes of Motility Suppression and c-di-GMP Production
Source: mBio. 2019 Apr 23;10(2):e00670-19. doi: 10.1128/mBio.00670-19 (PMC6479008; doi:10.1128/mBio.00670-19)
Supplement: TABLE S1 [file mBio.00670-19-st001.pdf]

Table S1. List of transposon insertion mutants.

Interrupted gene	Number of insertions	Predicted function
VCA0578	1	SgrR-like
VC0533	1	Lipoprotein NlpD
VCA0898	1	6-phosphogluconate dehydrogenase, decarboxylating
VC1718	1	Conserved Hypothetical protein
VC0374	1	Glucose-6-phosphate isomerase
VCA0016	1	1,4-alpha-glucan branching enzyme
Intergenic VC0305-VC0306	1	ATP-dependent RNA helicase RhlB (VC0305)<br>Thioredoxin (VC0306)
VC2751	1	Adenosine deaminase
VC1961	1	Cell division topological specificity factor

VC1375	1	Hypothetical protein
VC1907	1	Cys regulon transcriptional activator
VC0190	1	DNA helicase II
VC2064	1	Chemotaxis protein CheZ
VC0040	1	Hemolysin, putative
VC2203	2	Flagellar protein, FlgA
VC0547	1	Aspartokinase, alpha and beta subunits
VC0653	1	c-di-GMP phosphodiesterase A-related protein RocS
VCA0198	1	Site-specific DNA-methyltransferase, putative
VC2231	1	Oxidoreductase, acyl-CoA dehydrogenase family

VC2139	1	Flagellar rod protein FlaI
VC2062	1	Protein-glutamate methylesterase CheB
VC0239	1	Putative glycosyltransferase (LPS biosynthesis)
VC0241 (*manC*)	1	Mannose-1-phosphate guanylyltransferase (Perosamine biosynthesis)
VC0242 (*manB*)	1	Phosphomannomutase (Perosamine biosynthesis)
VC0243 (*gmd*)	1	GDP-mannose 4,6-dehydratase<br>(Perosamine biosynthesis)
VC0245 (*wbeG*)	1	Glycosyl transferase (Perosamine biosynthesis)
VC0246 (*wzm*)	1	Lipopolysaccharide/O-antigen transport protein<br>(O-antigen transport)
VC0250- (*wbeM*)	1	Iron-containing alcohol dehydrogenase family protein<br>(Tetronate biosynthesis)
VC0259 (*wbeU*)	1	Glycosyltransferase (O-antigen biosynthesis)

VC0260- (*wbeV*)	2	Mannosyl-transerase (O-antigen biosynthesis)
VC0263- (*wbeW*)	1	Galactosyl-transferase (O-antigen biosynthesis)
VC0395- (*galU*)	1	UTP-glucose-1-phosphate uridylyltransferase (LPS and EPS precursor)
