# Supplementary material for: Functional Specialization in Vibrio cholerae Diguanylate Cyclases: Distinct Modes of Motility Suppression and c-di-GMP Production
Source: mBio. 2019 Apr 23;10(2):e00670-19. doi: 10.1128/mBio.00670-19 (PMC6479008; doi:10.1128/mBio.00670-19)
Supplement: TABLE S2 [file mBio.00670-19-st002.pdf]

Table S2. Quantification of flagellated cells from Electron Microscopy images.

Strain	Flagellated cells	Non-flagellated cells	Total	% Flagellated cells
WT	135	9	144	94
Δ*gmd*	124	7	131	95
Δ*cdgD*	138	10	148	93
Δ*cdgH*	73	5	78	94
