# Supplementary material for: Functional Specialization in Vibrio cholerae Diguanylate Cyclases: Distinct Modes of Motility Suppression and c-di-GMP Production
Source: mBio. 2019 Apr 23;10(2):e00670-19. doi: 10.1128/mBio.00670-19 (PMC6479008; doi:10.1128/mBio.00670-19)
Supplement: TABLE S3 [file mBio.00670-19-st003.pdf]

Table S3. Bacterial strains and plasmids used in this study.

Strain or plasmid	Relevant genotype	Source
E. coli strains		
S17-1 (λ*pir*)	*recA, thi, pro,* RP4-2-Tc::Mu-Km::Tn7 λ*pir* Tp^r Sm^r r_K^- m_K^+ π^+	(1, 2)
SM10 (λ*pir*)	*Thi, thr, leu, tonA, lacY, supE, recA,* RP4-2-Tc::Mu λ*pir* Km^r π^+	(1)
V. cholerae strains		
FY_VC_0001	*Vibrio cholerae* O1 El Tor A1552, wild type, Rif^r	(3)
FY_VC_0352	FY_VC_0001 Δ*cdgD* Rif^r	(4)
FY_VC_1592	FY_VC_0001 Δ*cdgH* Rif^r	(5)
FY_VC_10970	FY_VC_0001 Δ*wavA* Rif^r	This study
FY_VC_10972	FY_VC_0001 Δ*gmd* Rif^r	This study
FY_VC_11017	FY_VC_10972 Δ*gmd*::*gmd* Rif^r	This study
FY_VC_11018	FY_VC_10972 Δ*gmd*::*gmd**STOP Rif^r	This study
FY_VC_10977	FY_VC_0352 Δ*gmd* Rif^r	This study
FY_VC_10982	FY_VC_1592 Δ*gmd* Rif^r	This study
FY_VC_12722	FY_VC_0001 Δ*flrB* (VC2136) Rif^r	A. Rogers
FY_VC_4327	*Vibrio cholerae* O1 El Tor A1552, rugose variant Δ*vps*-I Δ*vps*-II, Rif^r	(6)
FY_VC_9893	FY_VC_0001 Δ*lacZ*::P*vpsL*-*lacZ* ΔVC2285, ΔVC1376, ΔVC1104, ΔVC2454, ΔVC1067, ΔVCA0074	(7)

FY_VC_0237	Fy_Vc_1 mTn7-GFP, Rif^r Gm^r	(8)
FY_VC_10561	Fy_Vc_0352 mTn7-GFP, Rif^r Gm^r	This study
FY_VC_10562	Fy_Vc_1592 mTn7-GFP, Rif^r Gm^r	This study
FY_VC_10563	Fy_Vc_10972 mTn7-GFP, Rif^r Gm^r	This study
FY_VC_10564	Fy_Vc_10977 mTn7-GFP, Rif^r Gm^r	This study
FY_VC_10565	FY_VC_10982 mTn7-GFP, Rif^r Gm^r	This study
Plasmids		
pSC189	Mariner based Transposon TnSC189, Ap^r, Km^r	(9)
pGP704-*sacB*28	pGP704 derivative, *mob*/*oriT sacB*, Ap^r	G. Schoolnik
pBAD/myc-His-B	Arabinose-inducible expression vector with C-terminal myc epitope and six-His tags	Invitrogen
pFY4536	pBAD/myc-His-B containing *cdgD*-mycHis	MacroLabs
pFY4333	pFY4536 *cdgD*-mycHis with AAEEF mutation in the GGEEF motif	This work
pFY4375	pBAD/myc-His-B containing *cdgH*-mycHis	MacroLabs
pFY4932	pGP704sacB-Δ*wavA*	This work
pFY4492	pGP704sacB-Δ*gmd*	This work
pFY4328	pGP704sacB-Δ*gmd*::*gmd*	This work
pFY4330	pGP704sacB-Δ*gmd*::*gmd**STOP	This work
pFY1122	pBBRlux-P*flaA*-*luxCADBE*	F. Rivera
pMCM11	pGP704::mTn7-GFP, Gm^r, Ap^r	G. Schoolnik

pUX-BF13	oriR6K helper plasmid, mob/oriT, provides Tn7 transposition function in trans, Ap^r	(10)
pMMB67EH (Gm)	Expression vector derivative of pMMB67EH expressing *lacI* and containing a Ptac promoter, Gm^r.	Samuel Miller
pFY4357	pMMB67EH (Gm) containing the c-di-GMP biosensor, Gm^r	This study
pFY4535	pFY4357 containing the *hok*/*sok* region from pXB300, Gm^r	This study

Bibliography

1. Simon R, Priefer U, Pühler A. 1983. A Broad Host Range Mobilization System for In Vivo Genetic Engineering: Transposon Mutagenesis in Gram Negative Bacteria. Bio/Technology 1:784–791.

2. de Lorenzo V, Timmis KN. 1994. Analysis and construction of stable phenotypes in gram-negative bacteria with Tn5- and Tn10-derived minitransposons. Methods Enzymol 235:386–405.

3. Yildiz FH, Liu XS, Heydorn A, Schoolnik GK. 2004. Molecular analysis of rugosity in a *Vibrio cholerae* O1 El Tor phase variant. Mol Microbiol 53:497–515.

4. Lim B, Beyhan S, Meir J, Yildiz FH. 2006. Cyclic-diGMP signal transduction systems in *Vibrio cholerae*: modulation of rugosity and biofilm formation. Mol Microbiol 60:331–48.

5. Beyhan S, Odell LS, Yildiz FH. 2008. Identification and characterization of cyclic diguanylate signaling systems controlling rugosity in *Vibrio cholerae*. J Bacteriol 190:7392–405.

6. Fong JCN, Syed KA, Klose KE, Yildiz FH. 2010. Role of *Vibrio* polysaccharide (vps) genes in VPS production, biofilm formation and *Vibrio cholerae* pathogenesis. Microbiology 156:2757–2769.

7. Townsley L, Yildiz FH. 2015. Temperature affects c-di-GMP signalling and biofilm formation in *Vibrio cholerae*. Environ Microbiol 17:4290–4305.

8. Tischler AD, Camilli A. 2004. Cyclic diguanylate (c-di-GMP) regulates *Vibrio cholerae* biofilm formation. Mol Microbiol 53:857–69.

9. Chiang SL, Rubin EJ. 2002. Construction of a mariner-based transposon for epitope-tagging and genomic targeting. Gene 296:179–85.

10. Bao Y, Lies DP, Fu H, Roberts GP. 1991. An improved Tn7-based system for the single-copy insertion of cloned genes into chromosomes of gram-negative bacteria. Gene 109:167–8.
