# Supplementary material for: Functional Specialization in Vibrio cholerae Diguanylate Cyclases: Distinct Modes of Motility Suppression and c-di-GMP Production
Source: mBio. 2019 Apr 23;10(2):e00670-19. doi: 10.1128/mBio.00670-19 (PMC6479008; doi:10.1128/mBio.00670-19)
Supplement: TABLE S4 [file mBio.00670-19-st004.pdf]

Table S4. Primers used in this work

Primer	Sequence 5'- 3'	Assemble d Product
seq5	TGCTGAAGAGCTTGGCGG	Arbitrary PCR
Arb1	GGCCACGCGTCGACTAGTACN NNNNNNNNNTACNG	round 1
seq3	GACTCTGGGGTACGCGTCTA	Arbitrary PCR
Arb4	GGCCACGCGTCGACTAGTAC	round 2
pGP704sacB_rev-XbaI	TCTAGAACCGGTGACGTC	
223A_fwd	gtgacgtcaccggttctagaGGCATTAA ACGGCGATAC	
223B_rev	tccgacccatATGACAATCCGGCAG AGAAG	
223C_fwd	ggattgtcatATGGGTCGGATTGAT ATG	pFY_4932
223D_rev	actagagggtaccagagctcGGTGTTT GGTCGCTTTATC	
pGP704sacB-MCS_fwd	GAGCTCTGGTACCCTCTAG	
pGP704sacB_rev-XbaI	TCTAGAACCGGTGACGTC	
243A_fwd	gtgacgtcaccggttctagaGTGGTGAC TAAGTCGGGTG	pFY_4492
243B_rev	cagaagctacTTTATTCATTGTTAAA TATCCAATTTCTTAATTAATTTA G	

243C_fwd	caatgaataaaGTAGCTTCTGATTTAGAACAAG	
243D_rev	actagagggtaccagagctcTGCCATTAACAGCAGTAC	
pGP704sacB-MCS_fwd	GAGCTCTGGTACCCTCTAG	
gmd-knockIN_fwd	tctggatccacgaagcttcccatggGTGGTGACTAAGTCGGGTG	pFY_4328
gmd-knockIN_rev	gactagagggtaccagagctcTGCCATTAACAGCAGTACC	
gmd-stop_F	ATGATTAATCACAGGCATTACTG	pFY_4330
gmd-stop_R	TATCATTTATTCATTGTTAAATATCCAATTTC	
*cdgD*_AAEEF_F	GCACGGATTGCCGCAGAAGAGTTTG	pFY4333
*cdgD*_AAEEF_R	GATAAAATCCGATTCCCG	
FLAAPG2_SpeI	GGACTAGTTATGAGCGAAGTGAGTTGAG	pFY1122
*flaA*pshort_BamHI	CGCGGATCCTTAATGGTCATAGTTTGCTCTCC	
pMMB-Rv-III	CGCAACGCAATTAATGTAAG	pFY4357
pMMB-Fw-III	GAATTCGAGCTCGGTACC	
pMMB-hok-sok_R	CGCAACGTTCAAATCCGC	pFY4535
pMMB-hok-sok_F	TAAACTGCCAGGCATCAAATTAAG	

hok-sok_F	gagcggatttgaacgttgcgAACAAACT CCGGGAGGCAG
hok-sok_R	atttgatgcctggcagtttaACAACATCA GCAAGGAGAAAGG
