# Supplementary material for: Functional Specialization in Vibrio cholerae Diguanylate Cyclases: Distinct Modes of Motility Suppression and c-di-GMP Production
Source: mBio. 2019 Apr 23;10(2):e00670-19. doi: 10.1128/mBio.00670-19 (PMC6479008; doi:10.1128/mBio.00670-19)
Supplement: TEXT S1 [file mBio.00670-19-s0001.docx]

**Supplementary Materials**

**Extended Methods**

**Single-cell analysis of near surface motility behaviors by high speed microscopy**

*Image Processing*. After background subtraction, the gray scale images were converted to binary image. Adjacency tracking was done by connecting overlapping objects in consecutive frames. In case no overlapping was found, then nearby objects with the least distance below a threshold were connected. The minimum number of frames for a trajectory of a cell is 50, i.e. an object must be present for greater than 0.25 s to be considered as a cell. A run yields several hundred to several thousand trajectories depending on the run time and the characteristics of the mutant. Mutants that have less interaction with surface typically yield more trajectories.

*Detection of cell movement*. First of all, stalled cells (neither swimming nor attached to surface) are filtered and removed using a combination of radius of gyration (ROG), mean square displacement (MSD) slope, and other metrics. The free-swimming to surface-binding transition of *V. cholerae* cells are characterized by swimming speed, landing probability, surface residence time and quiescence interval. Swimming is defined by the following criteria: 1) the overall radius of gyration for the trajectory is greater than 5 pixels (1 µm), and 2) the instantaneous speed for greater than 95% of the trajectory is greater than 0.5 pixels/frame (20 µm/s), i.e. there is no long “pause” in the trajectory. For a given trajectory, the mean trajectory speed is the average of all frame-to-frame speeds. Landing of *V. cholerae* cells on surface is characterized with a sudden decrease of speed and radius of gyration in the trajectory. To detect landing, a 5-point window is used to measure the instantaneous radius of gyration (iROG) for a given trajectory, and continuous regions with iROG less than 0.15 µm (0.75 pixel) are found. Regions that are greater than 50 frames (0.25 second) are defined as surface landing. Landing probability is used to measure how much distance the cells swim before landing on surface. For each landing event, the swimming distances of all cells up to that time are tallied; the accumulated swimming distances vs landing are plotted and a linear fit is applied. Because the landing behaviors change over time, only the initial linear segment with sufficient number of landing events (>10) is used for linear fit (Fig.S4). The landing probability is obtained as inverse of the linear fit slope. Higher landing probability indicates stronger surface interaction and quicker surface attachment. For a given cell population, as more cells begin landing, less cells are available for free swimming. Surface residence time (SRT) is defined as the duration of cell attachment to surface. SRT is measured by counting the number of frames between landing and detachment (or the end of the run). If a cell lands multiple times in its trajectory, it yields multiple SRTs. Quiescence interval is defined as periods with little or no movement between movements for surface-landed cells. To measure quiescence interval, a 5-frame rolling window is used to measure the iROG; any segment in which all iROGs are below 0.05 µm, which is at the same level as imaging noise, is designated as a quiescence interval. A landed cell often goes through multiple quiescence intervals during its residence on surface.

*Estimating errors using bootstrap sampling method*. To estimate standard errors in landing probability for WT, ∆*cdgD*, ∆*cdgH*, ∆*gmd*, ∆*cdgD* ∆*gmd* and ∆*cdgH* ∆*gmd*, the trajectories for each mutant are randomly sampled 10,000 times to obtain 500 trajectories, from which landing probability is extracted. Standard error is calculated from the 10,000 values for the randomly sampled landing probability. To estimate standard errors in the fraction of surface residence time (SRT) against total time (swimming & surface attaching) for WT, ∆*cdgD*, ∆*cdgH*, ∆*gmd*, ∆*cdgD* ∆*gmd* and ∆*cdgH* ∆*gmd*, the trajectories for each mutant are randomly sampling 10,000 times with a sampling size of 300 trajectories. For each group of 300 trajectories, surface residence times that were greater than each continuous time on surface (CTOS) thresholds were tallied, respectively, and converted to fractions against the total time. The mean and standard error were then calculated from 10,000 sampling for each CTOS threshold. Similar method is used to estimate standard errors in the fraction of quiescence intervals (QI) against total SRT, except that for each group of 300 randomly sampled trajectories, QIs that were greater than each threshold were tallied, respectively, and converted to fractions against total surface residence time. The mean and standard errors were then calculated from 10,000 sampling for each QI threshold.

**Generation of genetic constructs.**

Primer designed was done using NEBuilder Assembly Tool (New England Biolabs, Ipswich, MA) for isothermal assembly and NEBaseChanger for Q5 site directed mutagenesis. Genomic DNA of *V. cholerae* O1 El Tor A1552 strain was used as template for Polymerase Chain Reaction (PCR) unless indicated otherwise. An overlapping PCR method was used to generate in-frame deletion constructs and knock-in constructs using previously published methods (1). The final assembly was generated by isothermal assembly using NEBuilder HiFi DNA Assembly Master Mix (New England Biolabs, Ipswich, MA) following the instruction of the manufacturer. Three premature stop codons situated after the first three amino acids of Gmd were introduced by site directed mutagenesis into the *gmd* knock-in construct (pFY4328). Site directed mutagenesis was carried out with the Q5 site directed mutagenesis kit (New England Biolabs, Ipswich, MA) using as DNA template pFY4328. The complementation constructs pFY4536 and pFY4375 were generated by MacroLab (UC, Berkeley) using the cloning vector pBAD-mycHis. In these constructs *cdgD* and *cdgH* are expressed to produce myc-6xHis tagged recombinant proteins. Site directed mutagenesis of *cdgD* (pFY4333) was achieved using the Q5 site directed mutagenesis kit and pFY4536 as DNA template.

The sequence of the c-di-GMP biosensor used in this study was reported previously (2). We designed *in silico* assembly units based on the reported sequence and cloned the biosensor in the broad-host replication plasmid pMMB67EH (Gm^r^) using isothermal assembly. The assembly units of the c-di-GMP biosensor were synthesized as gBlocks by Integrated DNA Technologies (Coralville, Iowa), the sequence is available as Supplementary Data 1. The assembled product (pFY4357) contains all the elements of the original biosensor, two strong promoters (Pbe region) driving expression of the genes encoding AmCyan and TurboRFP, and three c-di-GMP riboswitches Bc3, Bc4 and Bc5, that modulate production of TurboRFP. Additionally, we inserted the *hok*-*sok* toxin/anti-toxin cassette previously shown to provide stability to plasmid pXB300 in the absence of antibiotic selection (3). The *hok*/*sok* cassette was PCR amplified from pXB300 and cloned between the termination signals *rrnB* T1 and *rrnB* T2 of pFY4357 to generate pFY4535.

**Growth curve analysis.**

Overnight cultures were diluted 1:200 in fresh LB and grown at 30°C with agitation (200 r.p.m) for 23 hours. The turbidity of the culture was measured (Optical Density at 600nm) in two-sided disposable polystyrene cuvettes (VWR Radnor, PA) using a Beckman DU 530 UV/Vis Spectrophotometer (Backman, Indianapolis IN) at the time points indicated in Fig. S1. At least three independent biological replicates were analyzed at each time point.

**Purification and visualization of lypopolysaccharide by hot aqueous-phenol extraction.**

Lypopolysaccharide purification by hot aqueous-phenol was performed following a protocol reported for Gram-negative bacteria (4)**.** LPS samples were analyzed by SDS-PAGE using a 15% polyacrylamide gel. Gels were run for approximately one hour at 70 mV, afterwards the voltage was increased to 100-110 mV and run until the front of the gel reached the bottom edge. The gel was store in fixing solution (50% methanol, 3% Acetic acid) over night. The gel was stained with using Pro-Q Emerald 300 kit following the instructions of the manufacturer.

**Auto-aggregation assays.**

Cultures (5 mL) were grown in LB over-night at 30ºC with agitation at 200 rpm in glass test tubes. Grown cultures were incubated statically at room temperature and pictures were taken after 0 and 2 hours.

**Supplementary Data 1.** Sequence of the c-di-GMP biosensor assembled into pMMB67EH (Gm).

>Pba-amcyan-Bc3-5-turborfp

CCCAAGCTTGGCAAAACAACTTGAAAAAAGTTGTTGACAAAAAAGAAGCTGAATGTTATATTAGTAAAGTCTCGATGTTTGTTGACAGTATAAAGTTAGAAACTTATAATGATAAGAATTTTAGAAAGTCTAGAGGAGGAATTTTCCATGGCTCTTTCAAACAAGTTTATCGGAGATGACATGAAAATGACCTACCATATGGATGGCTGTGTCAATGGGCATTACTTTACCGTCAAAGGTGAAGGCAGCGGGAAGCCATACGAAGGGACGCAGACCTCGACTTTTAAAGTCACCATGGCCAACGGTGGGCCCCTTGCATTCTCCTTTGACATACTATCTACAGTGTTCATGTATGGAAATCGATGCTTTACTGCGTATCCTACCAGTATGCCCGACTATTTCAAACAAGCATTTCCTGACGGAATGTCATATGAAAGGACTTTTACCTATGAAGATGGAGGAGTTGCTACAGCCAGTTGGGAAATAAGCCTTAAAGGCAACTGCTTTGAGCACAAATCCACGTTTCATGGAGTGAACTTTCCTGCTGATGGACCTGTGATGGCGAAGATGACAACTGGTTGGGACCCATCTTTTGAGAAAATGACTGTCTGCGATGGAATATTGAAGGGTGATGTCACCGCGTTCCTCATGCTGCAAGGAGGTGGCAATTACAGATGCCAATTCCACACTTCTTACAAGACAAAAAAACCGGTGACGATGCCACCAAACCATGCGGTGGAACATCGCATTGCGAGGACCGACCTTGACAAAGGTGGCAACAGTGTTCAGCTGACGGAGCACGCTGTTGCACATATAACCTCTGTTGTCCCTTTCTGATAAGGATCCACGATAAATAAATACCTATTTTTGGCACACTATTCGAAAGGATAGGTCGCAAAGCTAAGAGTCTAAAGTAATGAAAATTACTATGATAGTCTGGTTGCAGTTTGGATTTTCACACATAGTTGTATGTATGAAAATCGAAGAGGCAACCGGATTTTTTATTGTCTCAAAAAGAAAAAATAAATGGGCACACTATTCGAAAGGATAGGTCGCAAAGCTAAGAGTCTAAGGTAATGAAAATTACTATGATAGTCTGGTTGCAGTTTGGATTTTCACACATGTTGTGATGTATGAAAATCGAAAAGGCAACCAGGCTTTTTATTTTGTCGACAAAACAGATAGGAGGAATTTTCCATGGGAAGTGAATTGATTAAAGAAAATATGCATATGAAATTATATATGGAAGGAACTGTTAATAATCATCATTTCAAATGTACATCAGAGGGAGAAGGAAAACCATATGAGGGAACGCAAACTATGAAAATAAAGGTGGTAGAAGGAGGGCCTTTACCATTTGCATTCGATATTTTAGCTACGAGTTTTATGTATGGAAGTAAAGCATTTATTAATCATACTCAGGGGATACCGGATTTCTTTAAACAATCATTTCCTGAAGGATTTACATGGGAACGTATTACAACATATGAAGATGGGGGAGTATTAACGGCAACTCAAGATACGTCATTTCAAAATGGATGCATCATATACAATGTTAAAATCAATGGGGTTAACTTTCCATCTAATGGACCTGTGATGCAGAAGAAAACACGTGGGTGGGAAGCGAATACTGAGATGTTATATCCAGCTGATGGAGGGTTAAGAGGACATAGTCAAATGGCATTGAAATTAGTAGGAGGGGGATACTTACATTGTTCTTTTAAAACTACATATCGTTCTAAGAAACCAGCTAAAAACTTAAAGATGCCGGGGTTTCATTTTGTAGACCATAGATTAGAACGTATTAAAGAAGCGGATAAAGAAACATATGTTGAGCAACATGAAATGGCTGTAGCAAAATATTGTGATTTACCTAGTAAATTAGGGCATAGATAACTCGAGAAGCTTACTAGTGGTACCCC

gBlocks used to assemble Pba-amcyan-Bc3-5-turborfp into pMMB67EH (Gm)

>gBlock-1

TCGCGCTAACTTACATTAATTGCGTTGCGCCCCAAGCTTGGCAAAACAACTTGAAAAAAGTTGTTGACAAAAAAGAAGCTGAATGTTATATTAGTAAAGTCTCGATGTTTGTTGACAGTATAAAGTTAGAAACTTATAATGATAAGAATTTTAGAAAGTCTAGAGGAGGAATTTTCCATGGCTCTTTCAAACAAGTTTATCGGAGATGACATGAAAATGACCTACCATATGGATGGCTGTGTCAATGGGCATTACTTTACCGTCAAAGGTGAAGGCAGCGGGAAGCCATACGAAGGGACGCAGACCTCGACTTTTAAAGTCACCATGGCCAACGGTGGGCCCCTTGCATTCTCCTTTGACATACTATCTACAGTGTTCATGTATGGAAATCGATGCTTTACTGCGTATCCTACCAGTATGCCCGACTATTTCAAACAAGCATTTCCTGACGGAATGTCATATGAAAGGACTTTTACCTATGAAGATGGAGGAGTTGCTACAGCCAGTTGGGAAATAAGCCTTAAAGGCAACTGCTTTGAGCACAAATCCACGTTTCATGGAGTGAACTTTCCTGCTGATGGACCTGTGATGGCGAAGATGACAACTGGTTGGGACCCATCTTTTGAGAAAATGACTGTCTGCGATGGAATATTGAAGGGTGATGTCACCGCGTTCCTCATGCTGCAAGGAGGTGGCAATTACAGATGCCAATTCCACACTTCTTACAAGACAAAAAAACCGGTGACGATGCCACCAAACCATGCGGTGGAACATCGCATTGCGAGGACCGACCTTGACAAAGGTGGCAACAGTGTTCAGCTGACGGAGCACGCTGTTGCACATATAACCTCTGTTGTCCCTTTCTGATAAGGATCCACGATAAATAAATACCTATTTTTGGCACACTATTCGAAAGGATAGGTCGCAAAGCTAAGAGTCTAAAGTAATGAAAATTACTATGATAGTCTGGTTGCAGTTTGGATTTTCACACATAGTTGTATGTATGAAAATCGAAGAGGCAAC

>gBlock-2

AGTTGTATGTATGAAAATCGAAGAGGCAACCGGATTTTTTATTGTCTCAAAAAGAAAAAATAAATGGGCACACTATTCGAAAGGATAGGTCGCAAAGCTAAGAGTCTAAGGTAATGAAAATTACTATGATAGTCTGGTTGCAGTTTGGATTTTCACACATGTTGTGATGTATGAAAATCGAAAAGGCAACCAGGCTTTTTATTTTGTCGACAAAACAGATAGGAGGAATTTTCCATGGGAAGTGAATTGATTAAAGAAAATATGCATATGAAATTATATATGGAAGGAACTGTTAATAATCATCATTTCAAATGTACATCAGAGGGAGAAGGAAAACCATATGAGGGAACGCAAACTATGAAAATAAAGGTGGTAGAAGGAGGGCCTTTACCATTTGCATTCGATATTTTAGCTACGAGTTTTATGTATGGAAGTAAAGCATTTATTAATCATACTCAGGGGATACCGGATTTCTTTAAACAATCATTTCCTGAAGGATTTACATGGGAACGTATTACAACATATGAAGATGGGGGAGTATTAACGGCAACTCAAGATACGTCATTTCAAAATGGATGCATCATATACAATGTTAAAATCAATGGGGTTAACTTTCCATCTAATGGACCTGTGATGCAGAAGAAAACACGTGGGTGGGAAGCGAATACTGAGATGTTATATCCAGCTGATGGAGGGTTAAGAGGACATAGTCAAATGGCATTGAAATTAGTAGGAGGGGGATACTTACATTGTTCTTTTAAAACTACATATCGTTCTAAGAAACCAGCTAAAAACTTAAAGATGCCGGGGTTTCATTTTGTAGACCATAGATTAGAACGTATTAAAGAAGCGGATAAAGAAACATATGTTGAGCAACATGAAATGGCTGTAGCAAAATATTGTGATTTACCTAGTAAATTAGGGCATAGATAACTCGAGAAGCTTACTAGTGGTACCCCGAATTCGAGCTCGGTACCCGGGGATCCTCT

**References**

1. Lim B, Beyhan S, Meir J, Yildiz FH. 2006. Cyclic-diGMP signal transduction systems in *Vibrio cholerae*: modulation of rugosity and biofilm formation. Mol Microbiol 60:331–48.

2. Zhou H, Zheng C, Su J, Chen B, Fu Y, Xie Y, Tang Q, Chou S-H, He J. 2016. Characterization of a natural triple-tandem c-di-GMP riboswitch and application of the riboswitch-based dual-fluorescence reporter. Sci Rep 6:20871.

3. Bina XR, Wong EA, Bina TF, Bina JE. 2014. Construction of a tetracycline inducible expression vector and characterization of its use in *Vibrio cholerae*. Plasmid 76:87–94.

4. Davis MR, Goldberg JB. 2012. Purification and visualization of lipopolysaccharide from Gram-negative bacteria by hot aqueous-phenol extraction. J Vis Exp.
